# Exosomes derived from mesenchymal stem cells inhibit neointimal hyperplasia by activating the Erk1/2 signalling pathway in rats

**DOI:** 10.1186/s13287-020-01676-w

**Published:** 2020-06-08

**Authors:** Zhihui Liu, Chao Wu, Xinliang Zou, Weiming Shen, Jiacai Yang, Xiaorong Zhang, Xiaohong Hu, Haidong Wang, Yi Liao, Tao Jing

**Affiliations:** 1 Department of Cardiology, Southwest Hospital, Army Medical University (Third Military Medical University), Chongqing, 400038 China; 2 State Key Laboratory of Silkworm Genome Biology, The Institute of Sericulture and Systems Biology, Southwest University, Chongqing, China; 3 Department of Thoracic Surgery, Southwest Hospital, Army Medical University (Third Military Medical University), Chongqing, China; 4 Laboratory of Integrative Medicine, School of Basic Medical Sciences, Shanghai University of Traditional Chinese Medicine, Shanghai, China; 5 The Institute of Burn Research, South-West Hospital, Army Medical University (Third Military Medical University), Chongqing, China

**Keywords:** MSC-Exo, Endothelial cells, Restenosis, Re-endothelialization, Erk1/2 signalling pathway, Neointimal hyperplasia

## Abstract

**Background:**

Restenosis is a serious problem in patients who have undergone percutaneous transluminal angioplasty. Endothelial injury resulting from surgery can lead to endothelial dysfunction and neointimal formation by inducing aberrant proliferation and migration of vascular smooth muscle cells. Exosomes secreted by mesenchymal stem cells have been a hot topic in cardioprotective research. However, to date, exosomes derived from mesenchymal stem cells (MSC-Exo) have rarely been reported in association with restenosis after artery injury. The aim of this study was to investigate whether MSC-Exo inhibit neointimal hyperplasia in a rat model of carotid artery balloon-induced injury and, if so, to explore the underlying mechanisms.

**Methods:**

Characterization of MSC-Exo immunophenotypes was performed by electron microscopy, nanoparticle tracking analysis and western blot assays. To investigate whether MSC-Exo inhibited neointimal hyperplasia, rats were intravenously injected with normal saline or MSC-Exo after carotid artery balloon-induced injury. Haematoxylin-eosin staining was performed to examine the intimal and media areas. Evans blue dye staining was performed to examine re-endothelialization. Moreover, immunohistochemistry and immunofluorescence were performed to examine the expression of CD31, vWF and α-SMA. To further investigate the involvement of MSC-Exo-induced re-endothelialization, the underlying mechanisms were studied by cell counting kit-8, cell scratch, immunofluorescence and western blot assays.

**Results:**

Our data showed that MSC-Exo were ingested by endothelial cells and that systemic injection of MSC-Exo suppressed neointimal hyperplasia after artery injury. The Evans blue staining results showed that MSC-Exo could accelerate re-endothelialization compared to the saline group. The immunofluorescence and immunohistochemistry results showed that MSC-Exo upregulated the expression of CD31 and vWF but downregulated the expression of α-SMA. Furthermore, MSC-Exo mechanistically facilitated proliferation and migration by activating the Erk1/2 signalling pathway. The western blot results showed that MSC-Exo upregulated the expression of PCNA, Cyclin D1, Vimentin, MMP2 and MMP9 compared to that in the control group. Interestingly, an Erk1/2 inhibitor reversed the expression of the above proteins.

**Conclusion:**

Our data suggest that MSC-Exo can inhibit neointimal hyperplasia after carotid artery injury by accelerating re-endothelialization, which is accompanied by activation of the Erk1/2 signalling pathway. Importantly, our study provides a novel cell-free approach for the treatment of restenosis diseases after intervention.

## Introduction

Vascular injury caused by percutaneous transluminal angioplasty (PTA) or stenting results in neointimal hyperplasia (NIH) by inducing abnormal vascular smooth muscle cells (VSMC) proliferation and migration [1]. Currently, drug-eluting stents have markedly decreased restenosis rates, but delayed arterial healing and late stent thrombosis have emerged as major concerns [2]. Therefore, new approaches or tools are needed to prevent restenosis after vascular damage and, in parallel, functional reconstruction of endothelial cells (EC) may be an effective strategy to inhibit the occurrence of NIH.

Mesenchymal stem cells (MSC) are multipotent and capable of being easily isolated from a variety of tissues, such as bone marrow, adipose and cord blood [3]. In addition, it is well known that MSC can secrete proangiogenic and cytoprotective factors through cell-to-cell communication [4]. Based on these attractive properties, MSC are recognized as tissue-repairing cells that may provide safe and effective cardiovascular cell therapies [5]. Moreover, accumulating evidence from in vivo and in vitro studies suggested that some of these reparative effects were mediated by paracrine factors secreted by MSC [6–9]. Recently, the biological factors in conditioned medium, including exosomes and soluble factors, derived from MSC have been extensively studied in different diseases, such as liver injury [10], glioma [11] and cardiovascular disease [12]. Compared with MSC, exosomes derived from mesenchymal stem cells (MSC-Exo) are more stable and storable, have no risk of aneuploidy, a lower possibility of immune rejection following allogeneic administration, and may provide alternative therapies for a variety of diseases [13].

Exosomes, also known as extracellular vesicles, are typically between 30 and 150 nm in diameter and contain a variety of RNAs, proteins and lipids [14]. Exosomes are secreted by a range of cell types, including MSC, and are transported between cells to regulate intercellular connections under certain conditions [15, 16]. Currently, exosomes are easy to obtain by ultracentrifugation or using purification kits, and they have also been extensively studied in cardiovascular diseases. For example, Li et al. [17] found that exosomes derived from cardiac progenitor cells could promote H9C2 cell growth and survival through the activation of the Akt/mTOR signalling pathway. Similarly, a study into the role of exosomes in the progression of acute myocardial infarction employed exosomes derived from mouse embryonic stem cells and revealed that immediate intramyocardial injection could significantly improve endogenous repair and preserve cardiac function in a mouse infarction model [18]. In another study, Kong et al. [19] revealed that exosomes derived from endothelial progenitor cells could inhibit NIH after carotid artery injury in rats. Thus, exosomes may have the same crucial role as cells in the treatment of diseases. However, to date, MSC-Exo have rarely been studied in neointimal formation following artery damage. Here we investigated the roles of MSC-Exo in the formation of NIH and re-endothelialization after rat carotid artery injury.

Rapid re-endothelialization after vascular damage is significant in restoring normal vascular function, reducing vascular inflammation and preventing adverse remodelling and neointimal formation [20]. Therefore, the present study first explored the underlying molecular mechanisms by which MSC-Exo accelerate the proliferation and migration of EC in vitro. Subsequently, we established a model of rat carotid artery balloon-induced injury to assess the effects of MSC-Exo on neointimal formation in vivo. Taken together, our results demonstrated a novel mechanism by which MSC-Exo can inhibit NIH following rat carotid artery balloon-induced injury, which might be a promising approach or tool and provide potential treatment strategies for cardiovascular protection after intervention.

## Materials and methods

### Cell culture

Briefly, 4-week-old SD rats (Animal Research Center of the Third Army Medical University, Chongqing, China) were sacrificed by cervical dislocation, followed by aseptic collection of the tibia and femur. The medullary cavity was rinsed using mesenchymal stem cell medium (MSCM) to collect bone marrow cells. Bone marrow cells were cultured in MSCM containing 5% exosome-depleted foetal bovine serum (FBS), 1% mesenchymal stem cell growth supplement and a 1% penicillin/streptomycin (P/S) solution (7501, Sciencell, USA) at 37 °C with 5% CO_2_. Then, flow cytometry analysis was performed to select for the MSC that were characterized by positive expression of CD29 (FITC-labelled) and CD90 (PE-labelled) and negative expression of CD11b (eFluor® 450-labelled). EC derived from rat carotid artery were purchased from the Meixuan Company (Shanghai, China) and cultured in 1640 medium (SH30809, Hyclone, USA) supported with 10% exosome-depleted FBS (SV30087, Hyclone, USA) and 1% P/S solution at 37 °C with 5% CO_2_.

### Isolation and characterization of mesenchymal stem cell-derived exosomes

After 48 h of culture, the MSC culture medium was harvested and centrifuged at 300*g* for 10 min and 2000*g* for 15 min to remove residual cell debris. The supernatants were subsequently filtered using a 0.22-μm filter membrane to remove larger particles. Exosomes were isolated from the culture medium using the Exo Quick-TC Kit (EXOTC50A-1, System Biosciences, USA) according to the manufacturer’s instructions. The pelleted exosomes were resuspended in 200 μL of phosphate buffered saline solution (PBS) and quantified by BCA protein assay kit (R33200, Thermo Fisher, USA). Exosomes were then assessed by transmission electron microscopy (TEM) and nanoparticle tracking analysis (NTA), as per previously described protocols [21, 22]. Exosomes were further verified by western blot analysis of exosome-associated markers including CD81, CD63, HSP70, Calnexin and TSG101.

### Internalization of PKH67-labelled exosomes in EC

Purified exosomes were labelled with 2 μmol/L of the fluorescent dye PKH67 (MINI67, Sigma, Germany) by incubation for 5 min at room temperature. Ultracentrifugation was performed to remove any remaining free dye at 120,000*g* for 70 min, followed by two washes with PBS and ultracentrifugation. To analyse the ingestion of exosomes by EC, EC were incubated with PKH67-labelled exosomes for 6 h and then stained with Hoechst 33342 (C1025, Beyotime, China). The internalization of PKH67-labelled exosomes by EC was visualized using a fluorescence microscope (IX73, Olympus).

### Cell growth assay

Cell proliferation was assessed using cell counting kit-8 (CCK8) reagent (NQ646, Dojindo, Japan). Briefly, EC were seeded at 5 × 10^3^ cells/well into a 96-well plate. EC were then treated with culture medium derived from mesenchymal stem cells (MSC-CM), culture medium derived from endothelial cells (EC-CM), MSC-Exo, exosome-depleted mesenchymal stem cells culture medium (CM-Exo-free) MSC-Exo + DMSO (SHBH9944, Sigma, Germany), MSC-Exo + Erk1/2 inhibitor (10 μM) [23–25] (SCH772984, Selleck, USA) or PBS and incubated for 24 h, 48 h and 72 h according to previous studies. Ten micrograms/millilitre of MSC-Exo was specifically determined to treat the cells. Then, 10 μL of CCK8 solution was added into each well and incubated in dark for 2 h. The absorbance at 450 nm was detected using Microplate Reader.

### Cell migration

EC were seeded at 4 × 10^5^ cells/well into a 24-well plate and cultured for 24 h to reach a fusion rate of 80%. The cells were then scratched with a 200-μL sterile pipette tip. The culture medium was immediately removed and replaced with 1640 medium supplied with MSC-Exo, MSC-Exo + DMSO, MSC-Exo + Erk1/2 inhibitor or PBS. Ten micrograms/millilitre of MSC-Exo was specifically determined to treat the cells. To exclude the effects of proliferation, cells were pretreated with 1640 medium containing 10 μg/μL mitomycin. Subsequently, the wound was monitored under a phase-contrast microscope (Olympus, IX51, Japan), and the percentage of cell closure was calculated by measurements of the scratch width using Image-Pro Plus software.

### Immunofluorescence

The indicated cells were fixed, permeabilized, blocked and incubated overnight with primary anti-Ki67 antibody (1:200, Abcam, UK) at 4 °C. Subsequently, the cells were incubated with secondary antibodies (1:100, ZSGB, China) for 1 h and stained with 1× Hoechst 33324 for 5 min at room temperature. The cell slides were mounted with anti-fluorescence quencher (P0126, Beyotime, China) and observed under a fluorescence microscope. For paraffin sections, they were deparaffinized, blocked and then incubated with primary anti-CD31 (1:200, R&D) and anti-von Willebrand factor (1:200, vWF, Abcam) antibodies at 4 °C overnight. Then, the sections were incubated with secondary antibodies (1:100, ZSGB, China) and stained with 1× Hoechst 33324 at room temperature. Subsequently, the sections were observed under a fluorescence microscope.

### Western blot

EC and exosomes were lysed with ice cold 1× RIPA lysis buffer supplemented with a protease/phosphatase inhibitor cocktail (KGP2100, Keygen, China) and quantified using a BCA kit. A total of 50 μg of protein for each sample was resolved by standard SDS-PAGE electrophoresis and transferred to a PVDF membrane. After blocking with 1× TBS-T containing 5% non-fat milk, the PVDF membrane was incubated overnight at 4 °C with the following primary antibodies: anti-CD81 (1:1000, NOVUS, USA), anti-CD63 (1:1000, Abcam, UK), anti-TSG101 (1:1000, Abcam, UK), anti-HSP70 (1:1000, R&D, USA), anti-Calnexin (1:1000, Abcam, UK), anti-MMP2 (1:1000, R&D, USA), anti-MMP9 (1:1000, NOVUS, USA), anti-Vimentin (1:1000, Abcam, UK), anti-PCNA (1:1000, CST, USA), anti-CyclinD1 (1:1000, CST, USA), anti-Erk1/2 (1:1000, R&D, USA) and anti-P-Erk1/2 (1:1000, R&D, USA). Then, the PVDF membrane was incubated with a corresponding horseradish peroxidase-conjugated secondary antibody (ZSGB, Beijing, China) at room temperature for 1 h and detected in chemiluminescent solution (32106, Thermo Fisher, USA).

### Animal experiments

Old male rats weighing 300 to 400 g were anaesthetized with an intraperitoneal injection of 5% chloral hydrate (100 mg/kg). The hair on the ventral aspect of the neck region was shaved, and the area was swabbed with 75% ethyl alcohol to expose the right common carotid artery (CCA). After blunt dissection alongside the right CCA toward the carotid artery bifurcation, the right internal carotid artery (ICA) was separated and looped proximally and distally with 8-0 silk suture for temporary blood flow cessation. The CCA and external carotid artery (ECA) were temporarily occluded with artery clips. Subsequently, the ICA underwent transverse arteriotomy, and a 1.5-F balloon catheter was inserted through the ICA to the CCA. The balloon was inflated to 10.0 atm and was then slowly withdrawn by rotation. After removal of the catheter, the ICA was ligated near the bifurcation, and the artery clips in the ECA and CCA were removed. The skin was closed, and the rats were divided into sham operation, normal saline and MSC-Exo groups (*n* = 6/group). In the MSC-Exo group, 1 mL of 100 μg/mL exosomes were intravenously injected every 3 days, and the normal saline group received the same volume of normal saline.

### Microscopy of rat carotid artery re-endothelialization

On weeks 2 and 4, the rats were anaesthetized with 5% chloral hydrate and intravenously injected with 2% Evans blue (*n* = 6/group). After 10 min, the rats were euthanized by an overdose of chloral hydrate. The right CCA was harvested and washed with PBS to remove residual blood. The area of nonendothelialization was marked with blue, while the area of re-endothelialization was marked with white. The white area to total area ratio was calculated as an indicator of re-endothelialization. The images were observed and collected using a light microscope (Olympus).

### Histochemistry and immunohistochemistry

All three groups of rats were sacrificed on weeks 2 and 4 after the establishment of the carotid artery balloon-induced injury model. The right CCA was harvested and fixed in 4% paraformaldehyde. Then, the CCA samples were dehydrated and embedded in paraffin. The samples were sectioned into 8-μm-thick pieces, followed by staining with haematoxylin and eosin (H&E). The intimal to medial area ratio (I/M) was measured using the Image-Pro Plus software program (Media Cybernetics, Rockville, MD). For immunohistochemistry, the sections were deparaffinized, blocked and then incubated with anti-CD31 (1:200), anti-vWF (1:200) and anti-α-smooth muscle actin (1:200, α-SMA, CST) primary antibodies at 4 °C overnight. The CD31- and vWF-positive areas were calculated according the integrated optical density (IOD) of the yellow-brown stained area using Image-Pro Plus software.

### Statistical analysis

Statistical analyses were performed using SPSS.23 software. All data from 3 independent experiments are presented as the means ± SD. The Student *t* test was performed to compare 2 groups; comparisons among ≥ 3 groups were evaluated via 1–way ANOVA followed by the Bonferroni multiple comparison test. *P* < 0.05 was considered statistically significant.

## Results

### The identification of MSC

**Fig. 1:**
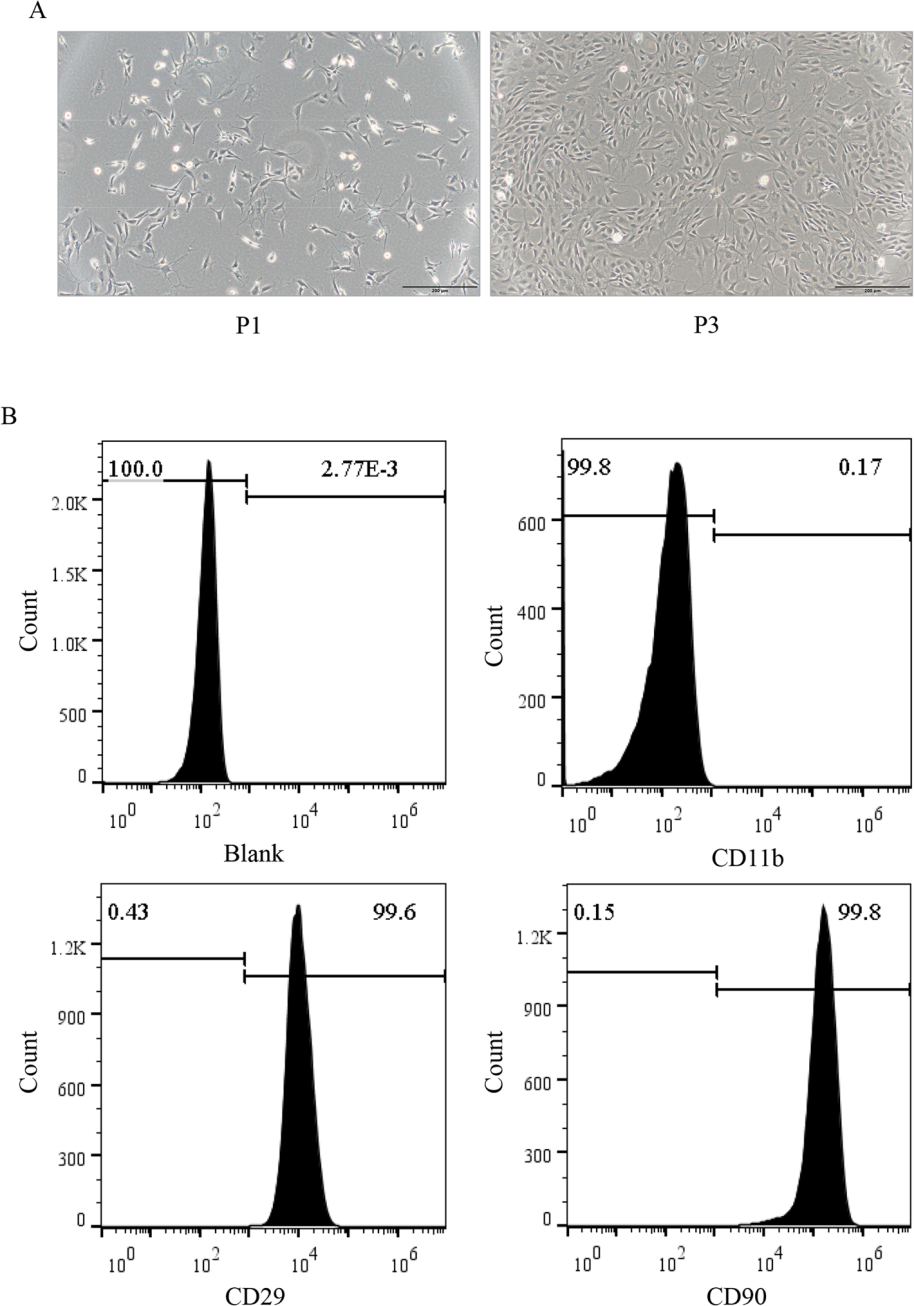
Characterization of MSC derived from rat bone marrow (RBM). **a** The morphology of the RBM-derived MSC from passage 1 (P1) and 3 (P3) (magnifications, × 100; scale, 50 μm). **b** Flow cytometry results showed that RBM-derived MSC were CD29- and CD90-positive, but CD11b-negative. All figures represent the average of three sets of independent experiments

The morphology of MSC was observed under a light microscope. Compared to that in the first generation, the morphology of the third generation MSC became spindle-shaped (Fig. 1a). MSC were cultured to the third generation, and the surface antigens CD11b, CD90 and CD29 on the MSC were detected by flow cytometry. The results showed that 0.17% of cells expressed the negative antigen CD11b, while the positive antigens CD29 and CD90 were expressed by 99.6% and 99.8% of cells, respectively, suggesting a high purity among the cultured MSC in our study (Fig. 1b).

### Characterization of mesenchymal stem cell-derived exosomes

**Fig. 2:**
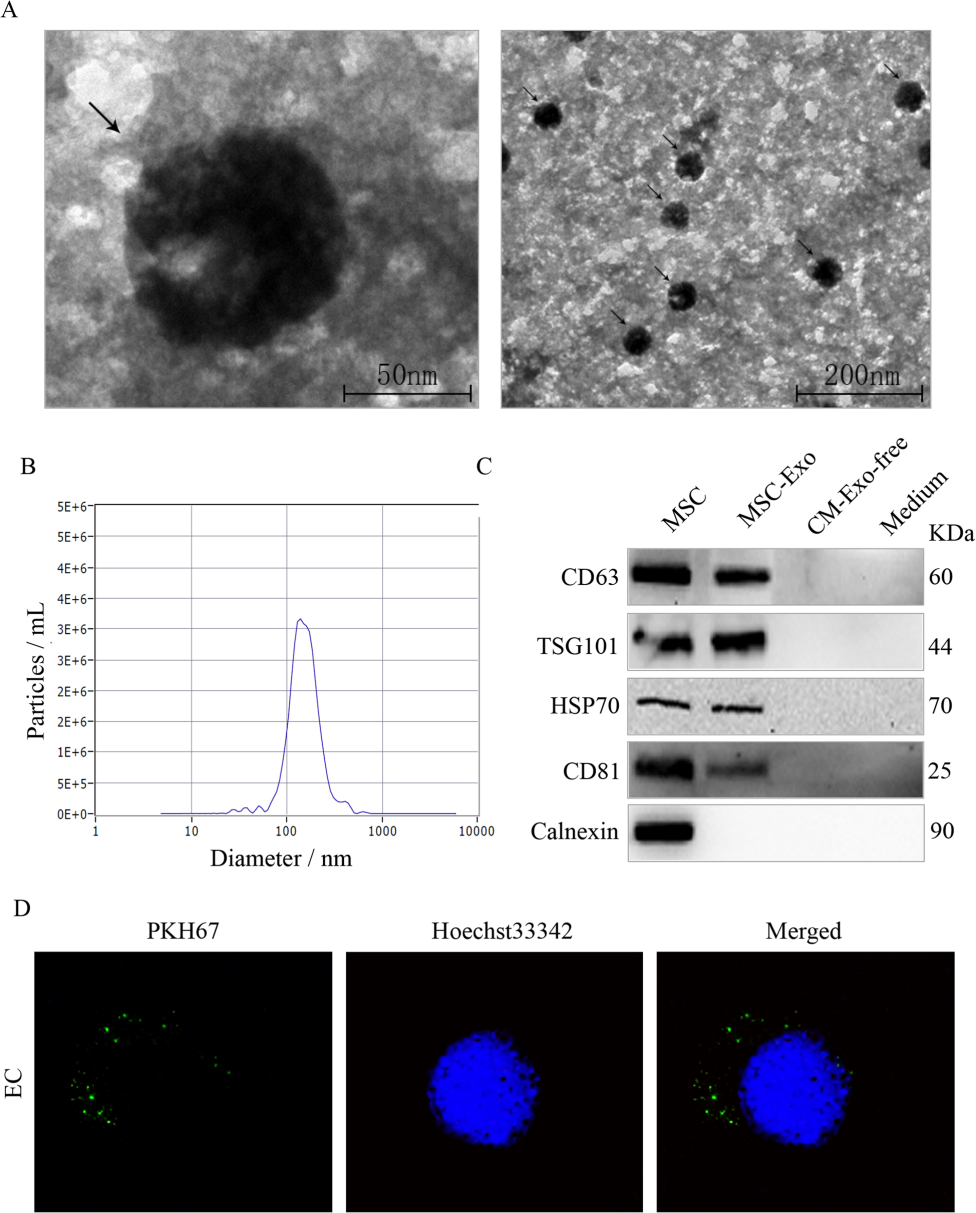
Characterization of MSC-Exo. **a** Transmission electron microscopy image of exosomes. The black arrows indicate exosomes. **b** Nanoparticle tracking analysis of the size of MSC-Exo. **c** Western blot analysis of the expression of characteristic exosomal proteins (CD63, TSG101, HSP70, CD81). **d** The internalization of PKH67-labelled MSC-Exo (green) by EC after 6 h of incubation (magnifications, × 1000). All figures represent the average of three sets of independent experiments

Transmission electron microscopy revealed that the isolated exosomes were round-shaped membrane vesicles (Fig. 2a). Particle size analysis showed that size of the exosome particles ranged from 30 to 150 nm (Fig. 2b). To further determine the properties of the exosomes, we measured the expression of exosome-associated protein markers by western blot. Our data showed that MSC and exosome particles expressed the specific proteins CD81, CD63, TSG101 and HSP70, while calnexin was only expressed in MSC (Fig. 2c). In addition, we observed that PKH67-labelled MSC-Exo were effectively internalized by EC and concentrated in the cytoplasmic region (Fig. 2d). These data indicated that the extracted exosomes are consistent with published standards and could be used in subsequent experiments.

### MSC-Exo promote the proliferation and migration of endothelial cells

**Fig. 3:**
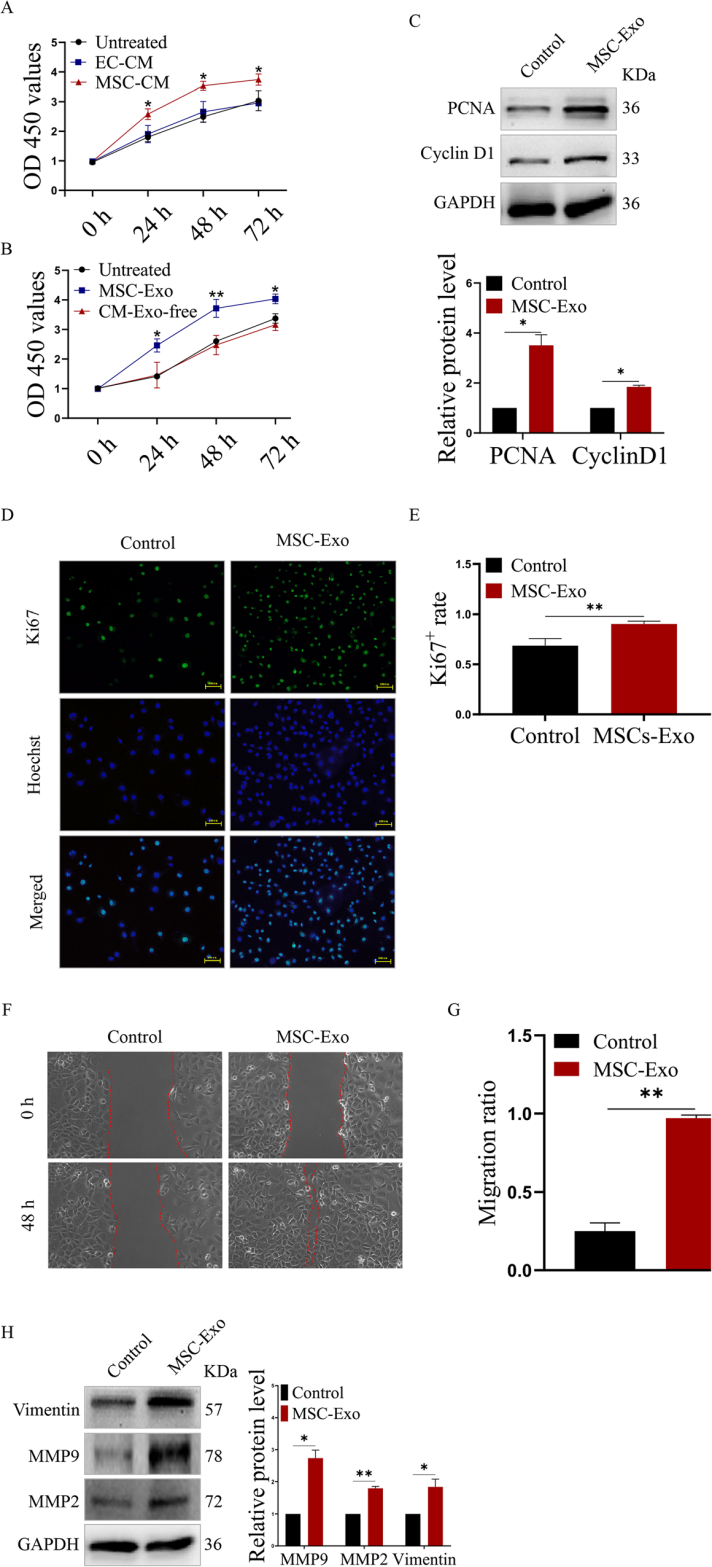
MSC-Exo accelerated the proliferation and migration of EC. **a** Cell growth analysis of MSC-CM-treated EC; EC-CM-treated EC served as the negative control group. **b** Cell growth analysis of MSC-Exo-treated EC; CM-Exo-free-treated EC served as the negative control group. **c** Western blot analysis of the expression of induced cell cycle-related proteins. **d** Immunofluorescence analysis of the number of Ki67-positive EC (green) after MSC-Exo treatment (magnifications, × 400; scale, 200 μm). **e** Quantitative analysis of the proportion of Ki67-positive EC. **f** Cell scratch experiment analysis of the migration of EC treated with MSC-Exo. **g** Quantitative analysis of the migration ratio of the indicated EC. **h** Western blot analysis of the expression of induced cell migration-related proteins. *P < 0.05 and **P < 0.01. All figures represent the average of three sets of independent experiments

To investigate whether exosomes have an effect on EC, we first measured the cell viability of EC that were treated with the MSC-CM, EC-CM, MSC-Exo and CM-Exo-free, with untreated EC serving as a control. Our data showed that MSC culture medium, as well as the exosomes extracted from MSC culture medium could significantly promote the growth of EC, while EC culture medium and exosome-deleted MSC culture medium had no effect on EC growth (Fig. 3a, b). Western blot analysis also demonstrated that, compared to the control EC group, the MSC-Exo-treated EC had upregulated expression of PCNA and CyclinD1, which are well-known cell cycle promoting markers (Fig. 3c). Moreover, immunofluorescence further showed that MSC-Exo significantly increased the number of Ki67 positive cells compared to the control EC group, with a percent increase from 68.66% to 90.33% (Fig. 3d, e). These results indicated that the MSC-Exo could promote the proliferation of EC. Further, we investigated whether MSC-Exo could also affect the migration of endothelial cells. Cell scratch experiments were performed in control EC and MSC-Exo-treated EC. Our results showed that MSC-Exo significantly promoted the healing of EC compared to that of the control EC, with a migration ratio increase from approximately 0.25 to 1 (Fig. 3f, g). Western blot analysis also verified that the expression of migration-promoting proteins such as Vimentin, MMP2 and MMP9 were also upregulated (Fig. 3h). These results suggested that MSC-Exo could also promote the migration of EC.

### MSC-Exo-induced proliferation and migration of endothelial cells requires Erk1/2 activity

**Fig. 4:**
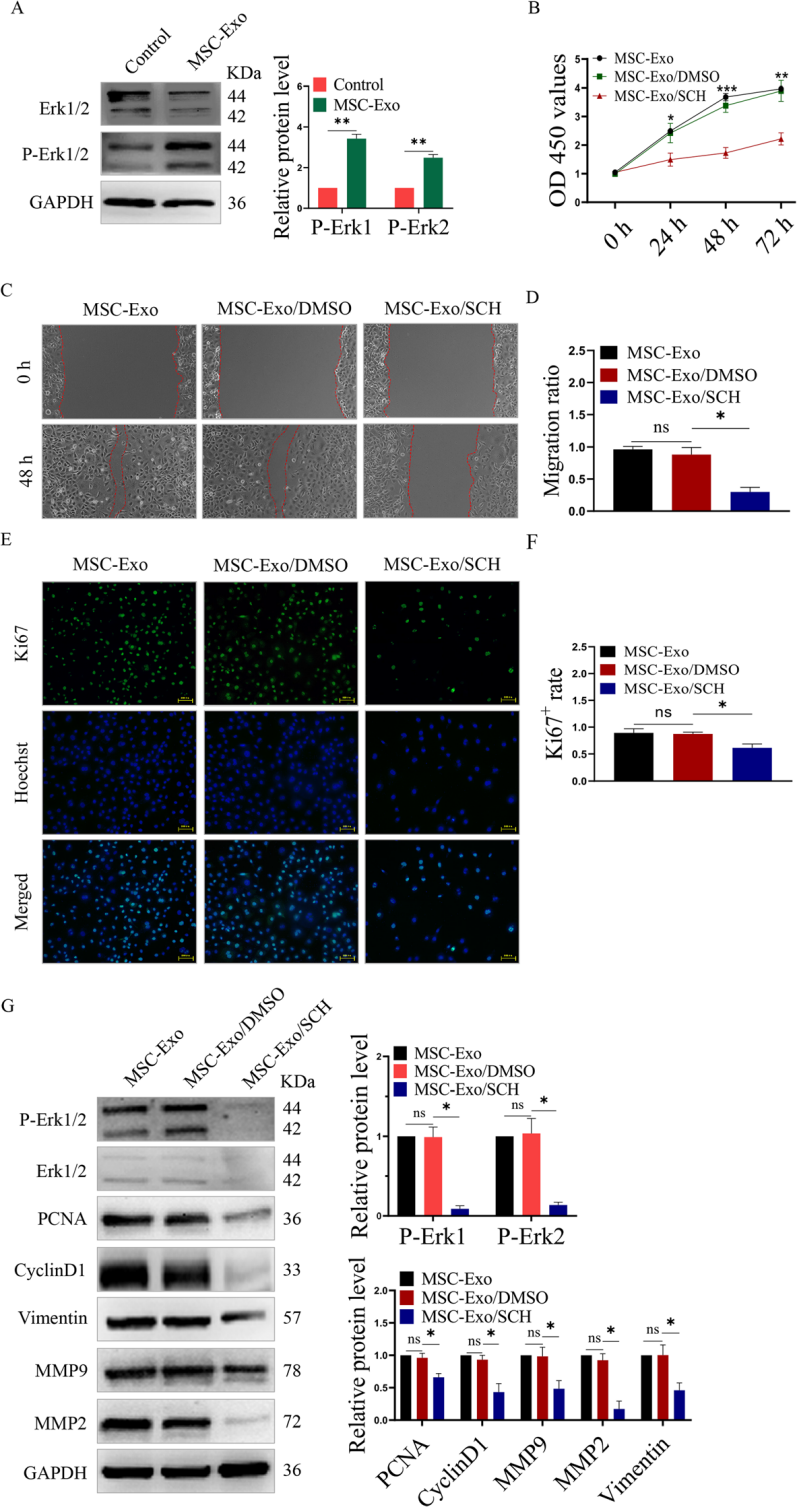
MSC-Exo activated the Erk1/2 signalling pathway. **a** Western blot analysis of the expression of Erk1/2 and phosphorylated Erk1/2. **b** Cell viability analysis of the growth of EC that were given combined treatment with MSC-Exo and SCH772984. **c** Cell scratch experiment analysis of the migration of EC that were given combined treatment of MSC-Exo and SCH772984. **d** Quantitative analysis of the migration ratio of the indicated EC. **e** Immunofluorescence analysis of the number of Ki67-positive EC (green) after treatment with MSC-Exo and SCH772984 (magnifications, × 400; scale, 200 μm). **f** Quantitative analysis of the proportion of Ki67-positive EC. **g** Western blot analysis of the expression of the indicated proteins. *P < 0.05, **P < 0.01 and ***P < 0.001. All figures represent the average of three sets of independent experiments

In our study, we found that MSC-Exo could induce the activation of the Erk1/2 signalling pathway in EC by increasing the phosphorylation of the Erk1/2 protein (Fig. 4a). Therefore, we suspected that the Erk1/2 signalling pathway may be involved in MSC-Exo-induced proliferation and migration of EC. To confirm our hypothesis, a specific Erk1/2 inhibitor, SCH772984 (SCH), which inactivates Erk1/2 by blocking phosphorylation [26], was used to determine the role of Erk1/2 activity in the regulation of MSC-Exo-induced proliferation and migration of EC. Our data indicated that SCH could suspend the MSC-Exo-induced the growth of EC (Fig. 4b) and suppress the MSC-Exo-induced migration of EC by reducing the migration ratio from approximately 1 to 0.3 (Fig. 4c, d). Additionally, immunofluorescence further demonstrated that SCH significantly reduced the percentage of Ki67-positive cells to 61.31% in MSC-Exo-treated EC (Fig. 4e, f). Moreover, western blot results showed that SCH inhibits the MSC-Exo-upregulated phosphorylation of the Erk1/2 protein, as well as the expression of downstream proliferation- and migration-related proteins, such as PCNA, CyclinD1, Vimentin, MMP2 and MMP9 (Fig. 4g). Together, these data suggested that MSC-Exo promoted the proliferation and migration of EC through the Erk1/2 signalling pathway.

### The effect of MSC-Exo on NIH after carotid injury in vivo

**Fig. 5:**
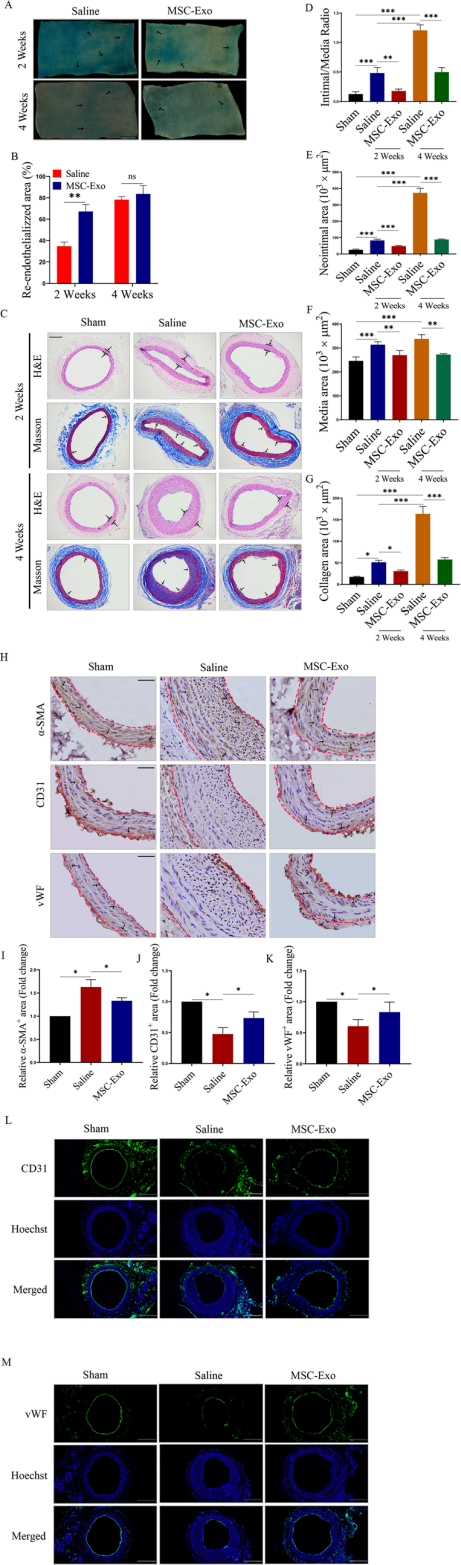
MSC-Exo inhibited NIH after carotid injury in vivo. **a** Evans blue staining analysis of the re-endothelialization of EC in the injured right CCA of the indicated rats at 2 weeks and 4 weeks (*n* = 6/group) (magnifications, × 40; scale, 200 μm). The black arrows indicate nonendothelialization area. **b** Quantitative analysis of the re-endothelialized areas of the indicated groups. **c** H&E and Masson staining analysis of the formation of neointimal hyperplasia in the indicated rats at 2 weeks and 4 weeks (*n* = 6/group) (magnifications, × 40; scale, 200 μm). The black arrows indicate collagen area. **d** Quantitative analysis of the intimal/media ratio, **e** the neointimal area, **f** the media area and **g** the collagen area of the indicated groups. **h** Immunohistochemical staining analysis of the expression of α-SMA, CD31 and vWF in the indicated rats (magnifications, × 100; scale, 50 μm). The black arrows indicate. Quantitative analysis of the relative **i** α-SMA^+^, **j** α-CD31^+^ and **k** vWF^+^ areas in the indicated rats; data were normalized to the sham group. Representative immunofluorescence images show the expression of **l** CD31 and **m** vWF in the indicated rats (magnifications, × 40; scale, 200 μm). *P < 0.05, **P < 0.01 and ***P < 0.001. All figures represent the average of three sets of independent experiments

After balloon injury, the injured right CCA of rats were harvested and the re-endothelialization was assessed by Evans blue staining (Fig. 5a). The re-endothelialization ratio of the MSC-Exo group was significantly higher than that of the saline control group after 2 weeks (34.70 ± 10.75% versus 70.28 ± 12.29%, *n* = 6, *P* < 0.05), while there was no significant difference in the re-endothelialization of the carotid artery after 4 weeks (78.33 ± 7.61% versus 83.70 ± 5.25%, *n* = 6, *P* = 0.32) (Fig. 5b). H&E and Masson staining was used to analyse NIH (Fig. 5c). The intimal/media (I/M) was measured as an index. The I/M in the MSC-Exo group was significantly reduced compared to that in the saline group at 2 weeks (0.48 ± 0.06% versus 0.18 ± 0.16%, *n* = 6, *P* < 0.01) and 4 weeks (1.21 ± 0.21 versus 0.50 ± 0.29, *n* = 6, *P* < 0.01) (Fig. 5d). The neointimal and media areas in the MSC-Exo group were both significantly reduced compared to that in the saline group at 2 weeks and 4 weeks (Fig. 5e, f), as well as the collagen area (Fig. 5g). Next, immunohistochemistry was used to examine the proliferation of EC and VSMC (Fig. 5h) after 4 weeks. Our data showed that the balloon injury increased the number of α-SMA-positive cells in the injured CCA; however, treatment with MSC-Exo could reduce this increase (Fig. 5i). Contrarily, compared to the sham control group, the CD31- and vWF-positive areas were significantly decreased in the saline group, while treatment with MSC-Exo reversed this decrease (Fig. 5j, k). In addition, immunofluorescence was used to examine the expression of CD31 and vWF. Our results showed that the treatment of saline could decrease the fluorescence intensity of CD31 and vWF compared to the sham group, while MSC-Exo significantly increased the fluorescence intensity of CD31 and vWF compared to that in the saline group after 4 weeks (Fig. 5l, m). Taken together, these data indicated that MSC-Exo could inhibit NIH by promoting vascular repair and re-endothelialization after vascular injury.

## Discussion

All types of intervention result in some degree of arterial injury, the response to which determines the success of the procedure and the long-term prognosis of the patient. Vascular injury is considered to be the main cause of neointimal hyperplasia initiation and progression [27]. Although current strategies to prevent restenosis are focused on the inhibition of neointimal hyperplasia through drug-eluting stents and vascular brachytherapy, restenosis rates following endovascular intervention remain high [22, 28]. Thus, it is important to understand the molecular mechanisms involved in neointima formation.

A previous study demonstrated that MSC derived from bone marrow could inhibit neointimal hyperplasia after artery injury [29]. They have shown promising effects in experimental models of artery injury and have been used in clinical practice for more than a decade. To date, although advanced stem cell transplantation technology has indeed had beneficial effects on the treatment of cardiovascular disease, the mechanism of this protective effect is generally unclear because of the low efficiency of transdifferentiation into damaged cells after cell transplantation [30]. Interestingly, it was found that MSC improve myocardial function through paracrine effects rather than direct differentiation into cardiomyocytes [12]. Importantly, it has also been reported that MSC have the ability to differentiate into a variety of cell types, such as endothelial cells, smooth muscle cells, myocardium, skeletal muscle and cartilage [31, 32]; however, the beneficial effects of MSC do not depend on their ability to differentiate and replace damaged tissue but are primarily mediated by the release of paracrine factors [33]. Thus, we hypothesized that MSC may affect the NIH of rats through a paracrine network.

In addition to paracrine molecules, a series of secretory membrane-encapsulated vesicles, especially exosomes that participate in a variety of physiological processes, are receiving increasing attention [34]. Exosomes, as a type of extracellular vesicles, originate from the endosomal bodies of cells; endosomes bud inward to form multivesicular bodies (MVBs), which are then fused with the plasma membrane and released into inner vesicles [35]. Generally, transmission electron microscopy, nanoparticle tracking analysis and western blots are used to assess the characterization of these particles. It is through the transfer of their contents, including proteins, lipids and nucleic acid [36], to target cells that exosomes exert functional effects. Increased levels of circulating exosomes have been observed during cardiovascular diseases, including myocardial infarction, and there is also evidence for a role of exosomes in atherosclerosis, neointima formation and vascular repair and remodelling. However, to date, research into the role of exosomes in disease has relied on exosomes isolated from cultured cells or blood, which may not reflect the in vivo situation. For example, in a rat model of balloon-induced carotid artery injury, exosomes derived from endothelial progenitor cells (EPC) were used to investigate neointima formation and vascular repair, revealing that attenuated vascular repair could enhance re-endothelialization in vivo and endothelial function in vitro [37]. Similarly, a study into the role of exosomes in the progression of atherosclerosis employed exosomes derived from oxidized low-density lipoprotein (oxLDL)-stimulated macrophages and revealed that intravenous administration of oxLDL-treated macrophage cell-derived exosomes into male ApoE-deficient atherosclerosis mice significantly deteriorated atherosclerosis in vivo [38]. In another study, MSC-Exo were shown to exert antiapoptotic and anti-inflammatory effects on intestinal ischaemia-reperfusion-induced lung damage; their effects were accompanied by the downregulation of TLR4 and NF-κB expression, which further protected the lungs against ischeamia-reperfusion-induced acute lung injury [39].

It has been known that the proliferation and migration of smooth muscle cells is exceedingly significant for the initiation and progression of neointimal hyperplasia, but the effects of endothelial cells have also been considered irreplaceable. However, to date, the precise mechanism of endothelial cell influence on the development of neointimal hyperplasia remains obscure. It has been reported that exosomes secreted by a variety of cell types may affect these processes. For example, exosomes derived from endothelial progenitor cells have been shown to limit neointimal hyperplasia and in a study by Kong et al. [19], this was attributed to decreased VSMC proliferation and accelerated re-endothelialization. In another study, Li et al. [40] demonstrated that EC-derived exosomes could repress platelet-derived growth factor (PDGF)-BB-induced phenotypic switching of VSMC and neointimal formation after carotid injury, while the activation of endothelial CD137 signalling attenuated their effects on VSMC. In addition, there is also evidence showing that exosomes derived from M1 macrophages may exert effects on neointimal hyperplasia via exosomal miRNAs. Exosomes derived from M1 macrophages were shown to aggravate neointimal hyperplasia by delivering miR-222 into VSMC [41]. Therefore, we hypothesized that MSC-Exo may be involved in the process of vascular endothelial cell regeneration and proliferation, which may further inhibit the progression of NIH after carotid injury in rats. Interestingly, our in vitro experiments strongly demonstrated that MSC-Exo could facilitate the proliferation and migration of EC.

Accumulating evidence suggested that MSC-Exo could exert effects via activating/inactivating signalling pathways including the TGF-β1 [42], NF-κB [39, 43] and Wnt [44] signalling pathways. However, to date, the Erk signalling pathway has rarely been studied with MSC-Exo. Furthermore, the Erk signalling pathway is a prototypic mitogen-activated protein kinase (MAPK) signalling cascade that is involved in cell survival, proliferation, migration and differentiation [45, 46]. Several studies also showed that the Erk signalling pathway was closely associated with the growth, survival and migration of cells, whereas these effects were reversed when the signalling pathway was blocked using corresponding inhibitors [47, 48]. Thus, we hypothesized that MSC-Exo could activate/inactivate the Erk signalling pathway during the proliferation and migration of EC. Our experiments further showed that MSC-Exo could promote the proliferation and migration of EC via the Erk1/2 signalling pathway.

Numerous studies have shown that restenosis following endovascular treatment represents an over-healing response to iatrogenic damage to the vessel wall during angioplasty and stenting, which is characterized by the apoptosis of EC and the adhesion and invasion of macrophages, as well as the proliferation and migration of smooth muscle cells [49, 50]. Thus, the accelerated re-endothelialization of the damaged vessels may be significant for the recovery of endothelial function to prevent the formation of a thrombus and excessive neointimal formation. Indeed, our in vivo experiments strongly demonstrated that MSC-Exo could inhibit neointimal formation via accelerated re-endothelialization and the inhibition of VSMC proliferation.

**Fig. 6:**
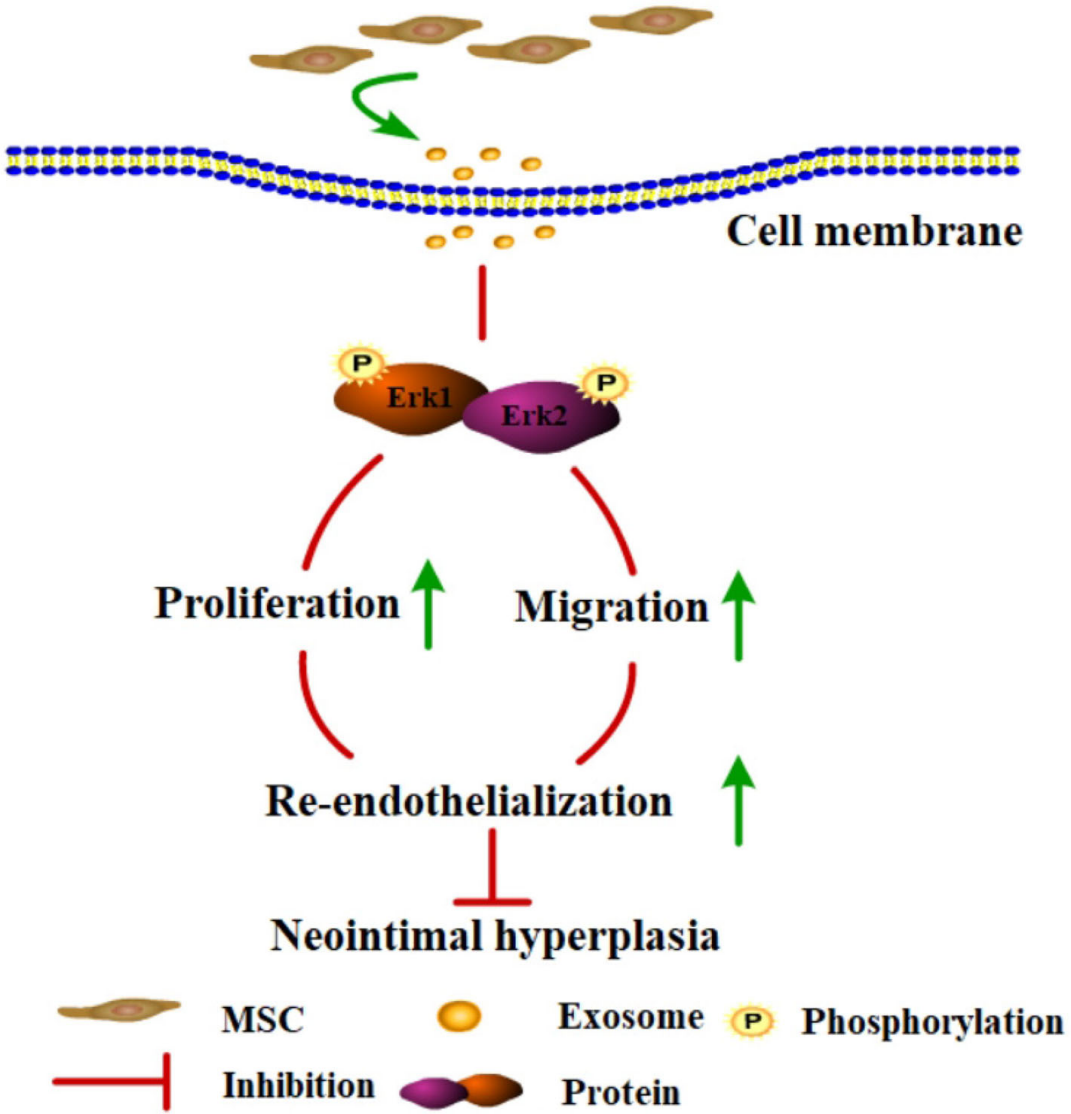
A working model for how MSC-Exo activation of the Erk1/2 signalling pathway inhibits neointimal hyperplasia via promoting re-endothelialization after vascular injury

In summary, the present study is the first to demonstrate that MSC-Exo could promote the proliferation and migration of EC, which further accelerated re-endothelialization through the activation of the Erk1/2 signalling pathway, suggesting that MSC-Exo could alleviate the progression of NIH through the Erk1/2 signalling pathway after carotid injury in rats (Fig. 6). However, there are certain limitations to the clinical application of MSC-Exo. Currently, we still face enormous challenges in utilizing exosomes for treatment. First, the minimum effective dose of exosomes needs to be optimized. Second, animal models in addition to the rat should be adopted, including large animal models, such as pigs, rabbits and monkeys. Third, exosome extraction methods need to be further optimized to obtain purer and higher volumes of exosomes. In future studies, we should pay more attention to the biological distribution and bioactivity of exosomes in vivo to determine the optimal treatment window and dosage.

## Conclusion

In this study, we have demonstrated that MSC-Exo promoted the proliferation and migration of EC through the Erk1/2 signalling pathway, which accelerated re-endothelialization, thus alleviating the progression of NIH after carotid injury in rats.

## Supplementary information

**Additional file 1.**

## Data Availability

The data and materials support that findings could be found.

## Abbreviations

CCA: Common carotid artery
CCK8: Cell counting kit-8
CM-Exo-free: Exosome-depleted mesenchymal stem cells culture medium
ECA: External carotid artery
EC: Endothelial cells
EC-CM: Culture medium derived from endothelial cells
EPC: Endothelial progenitor cells
FBS: Foetal bovine serum
H&E: Haematoxylin and eosin
ICA: Internal carotid artery
I/M: Intimal to medial area ratio
IOD: Integrated optical density
MAPK: Mitogen activated protein kinase
MSC: Mesenchymal stem cells
MSC-CM: Culture medium derived from mesenchymal stem cells
MSC-Exo: Exosomes derived from mesenchymal stem cells
MSCM: Mesenchymal stem cell medium
MVBs: Multivesicular bodies
NIH: Neointimal hyperplasia
NTA: Nanoparticle tracking analysis
oxLDL: Oxidized low-density lipoprotein
PBS: Phosphate buffered saline
PDGF: Platelet-derived growth factor
P/S: Penicillin/streptomycin
PTA: Percutaneous transluminal angioplasty
SCH: Erk1/2 inhibitor SCH772984
α-SMA: α-Smooth muscle actin
TEM: Transmission electron microscopy
VSMC: Vascular smooth muscle cells
vWF: von Willebrand factor
