# Supplementary material for: Exosomes derived from mesenchymal stem cells inhibit neointimal hyperplasia by activating the Erk1/2 signalling pathway in rats
Source: Stem Cell Res Ther. 2020 Jun 8;11:220. doi: 10.1186/s13287-020-01676-w (PMC7278178; doi:10.1186/s13287-020-01676-w)
Supplement: Supplementary file 1 — Additional file 1. [file 13287_2020_1676_MOESM1_ESM.pptx]

## Slide 1
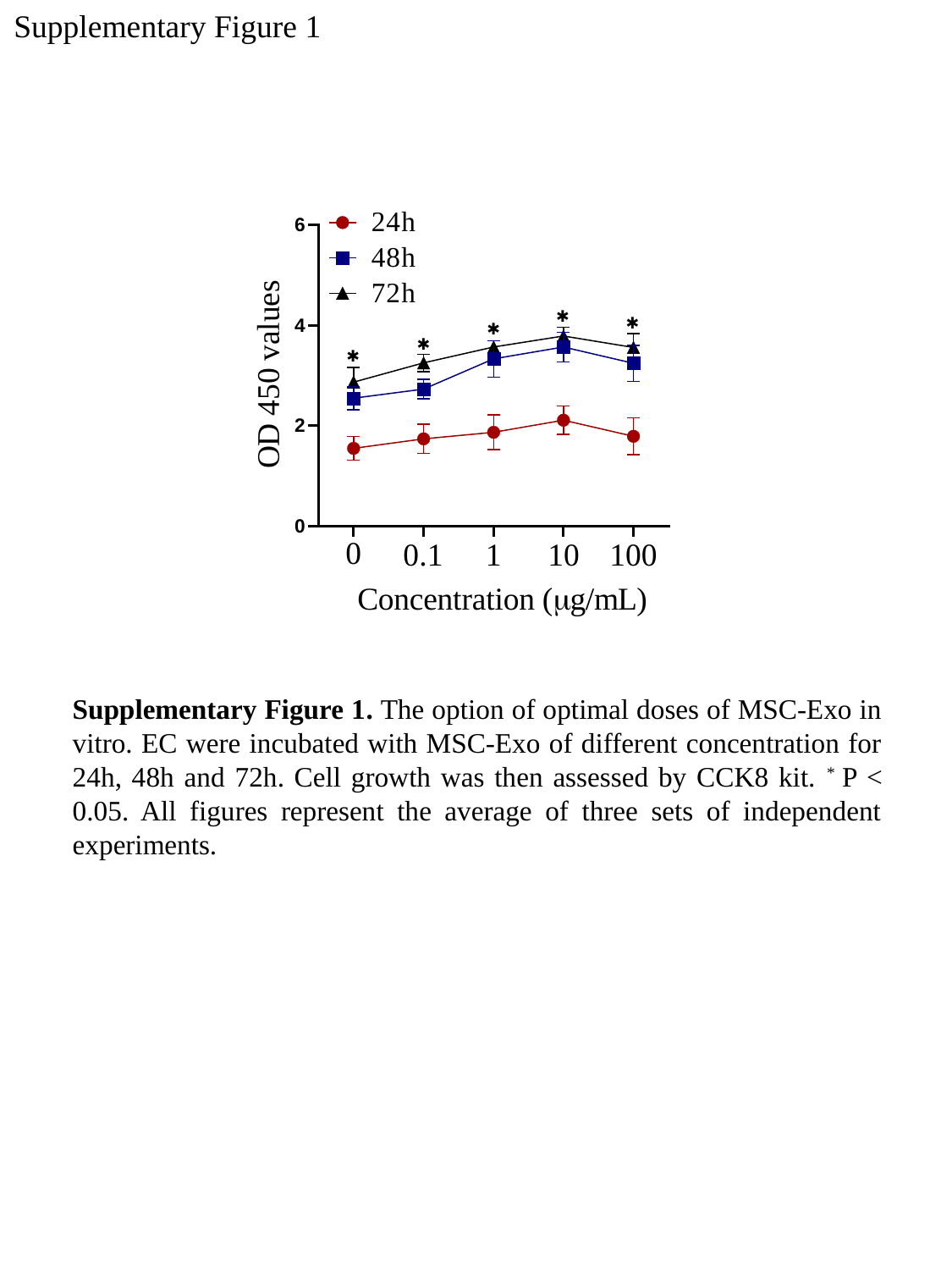

Supplementary Figure 1
Supplementary Figure 1. The option of optimal doses of MSC-Exo in vitro. EC were incubated with MSC-Exo of different concentration for 24h, 48h and 72h. Cell growth was then assessed by CCK8 kit. * P < 0.05. All figures represent the average of three sets of independent experiments.

## Slide 2
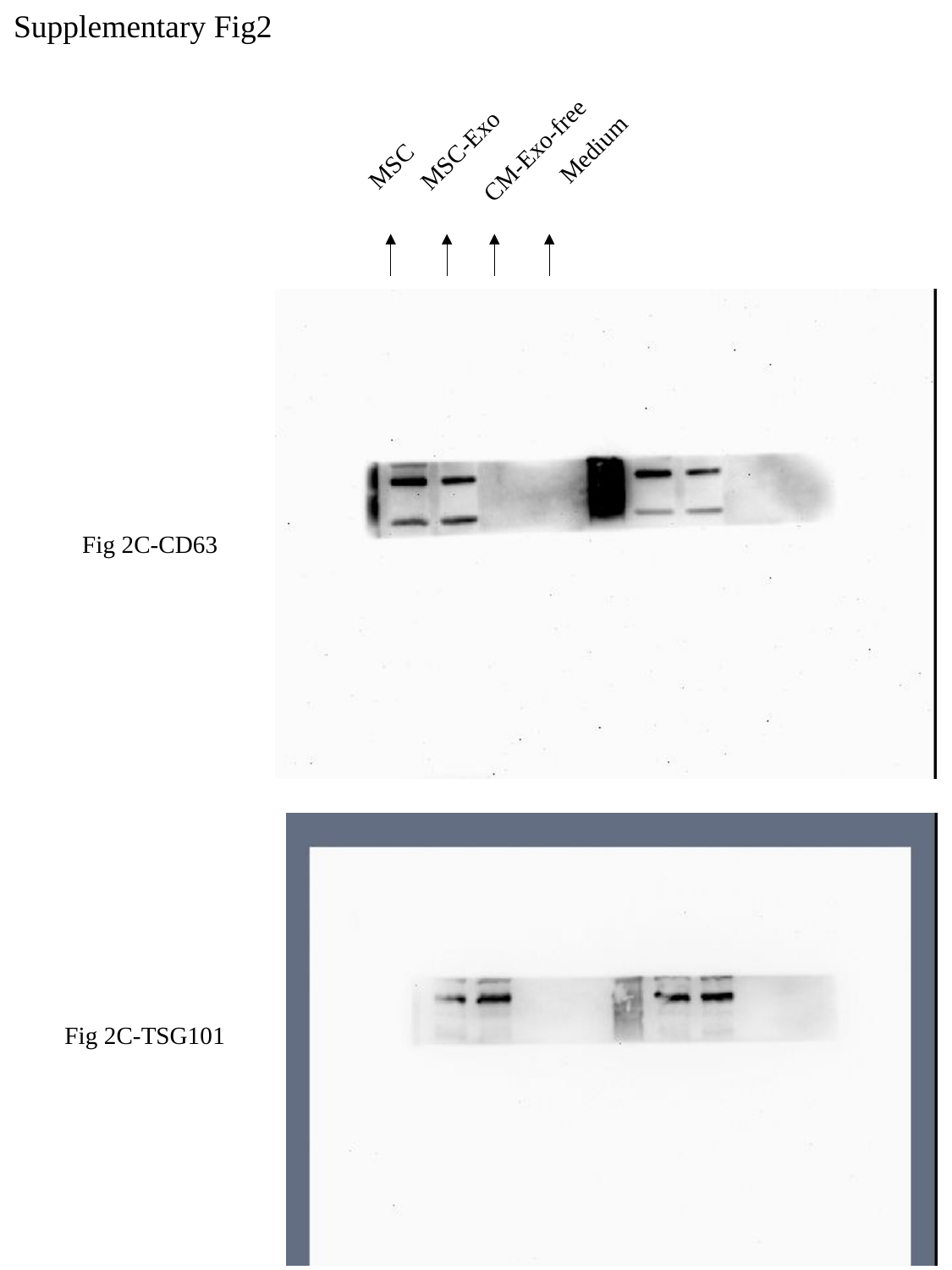

Supplementary Fig2
Medium
MSC-Exo
CM-Exo-free
MSC
Fig 2C-CD63
Fig 2C-TSG101

## Slide 3
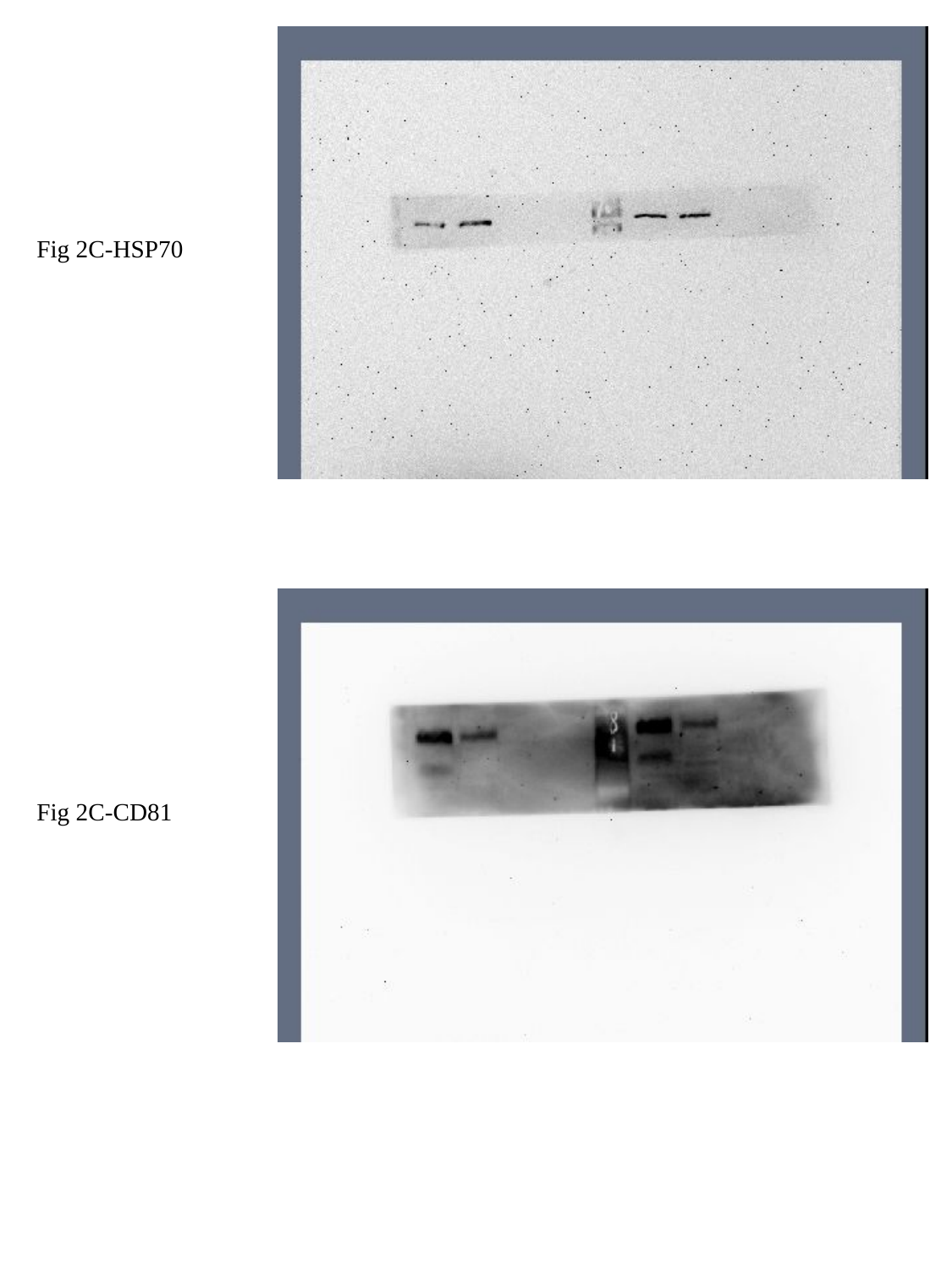

Fig 2C-HSP70
Fig 2C-CD81

## Slide 4
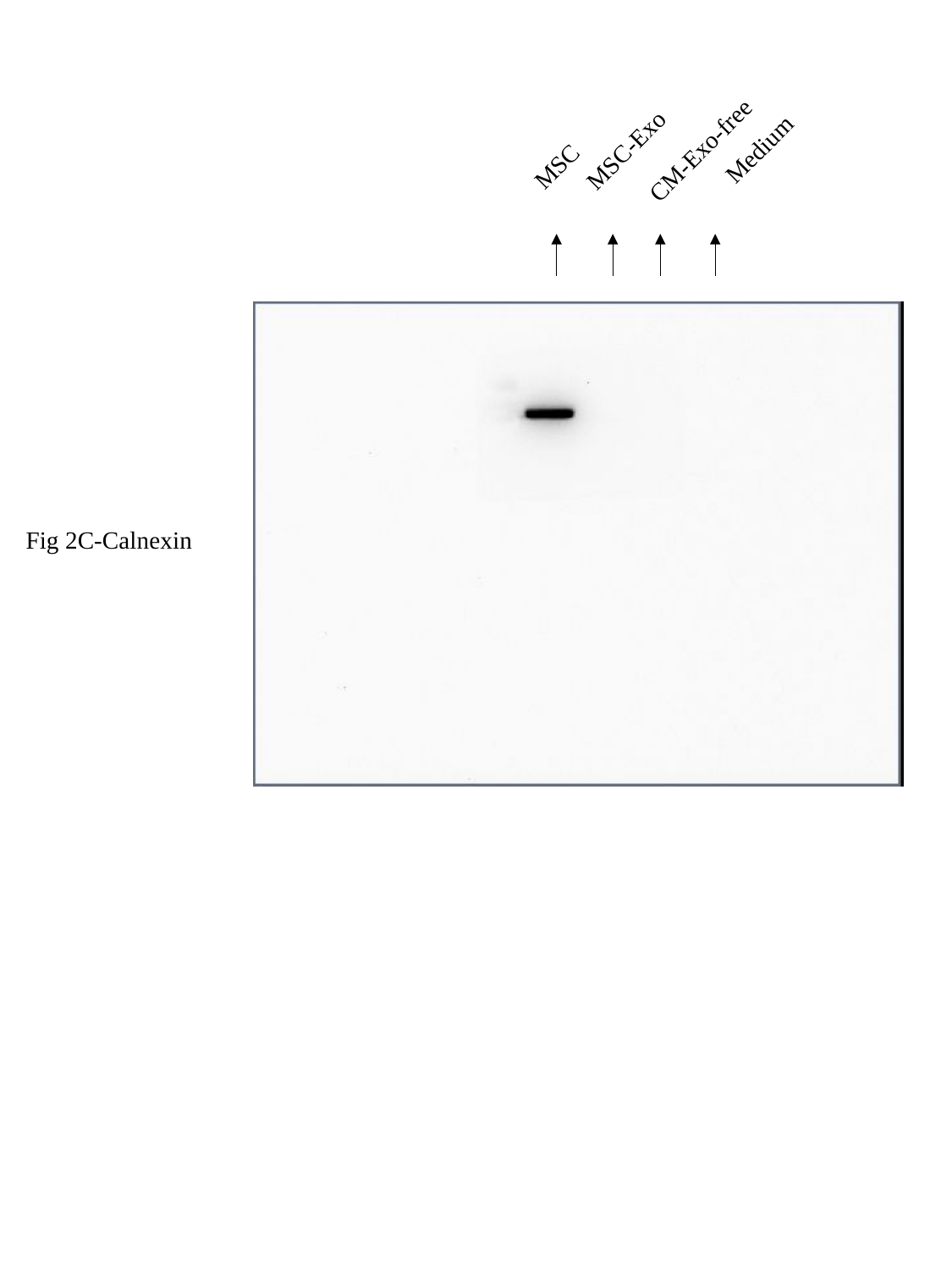

Medium
MSC-Exo
CM-Exo-free
MSC
Fig 2C-Calnexin

## Slide 5
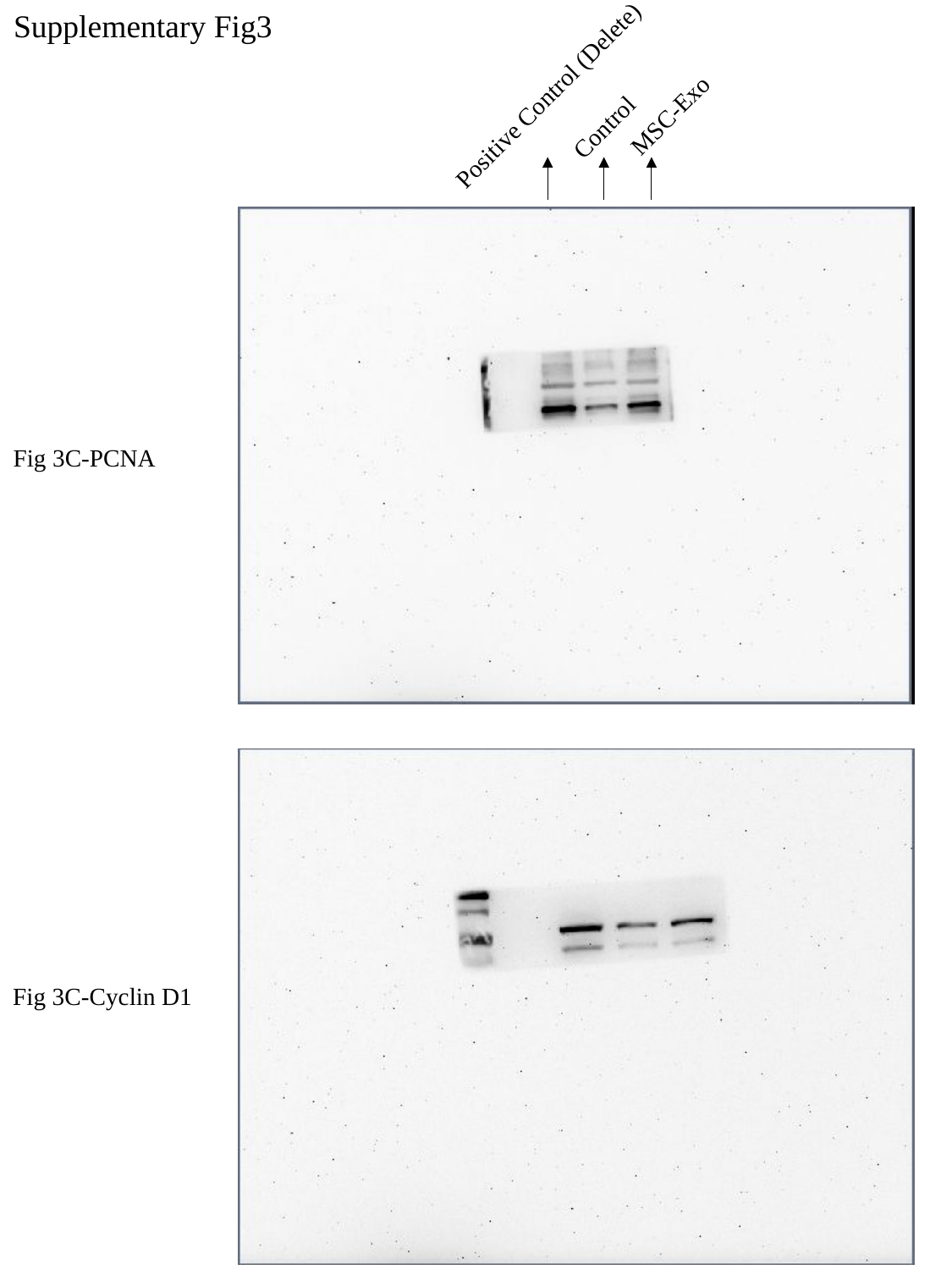

Supplementary Fig3
Positive Control (Delete)
MSC-Exo
Control
Fig 3C-PCNA
Fig 3C-Cyclin D1

## Slide 6
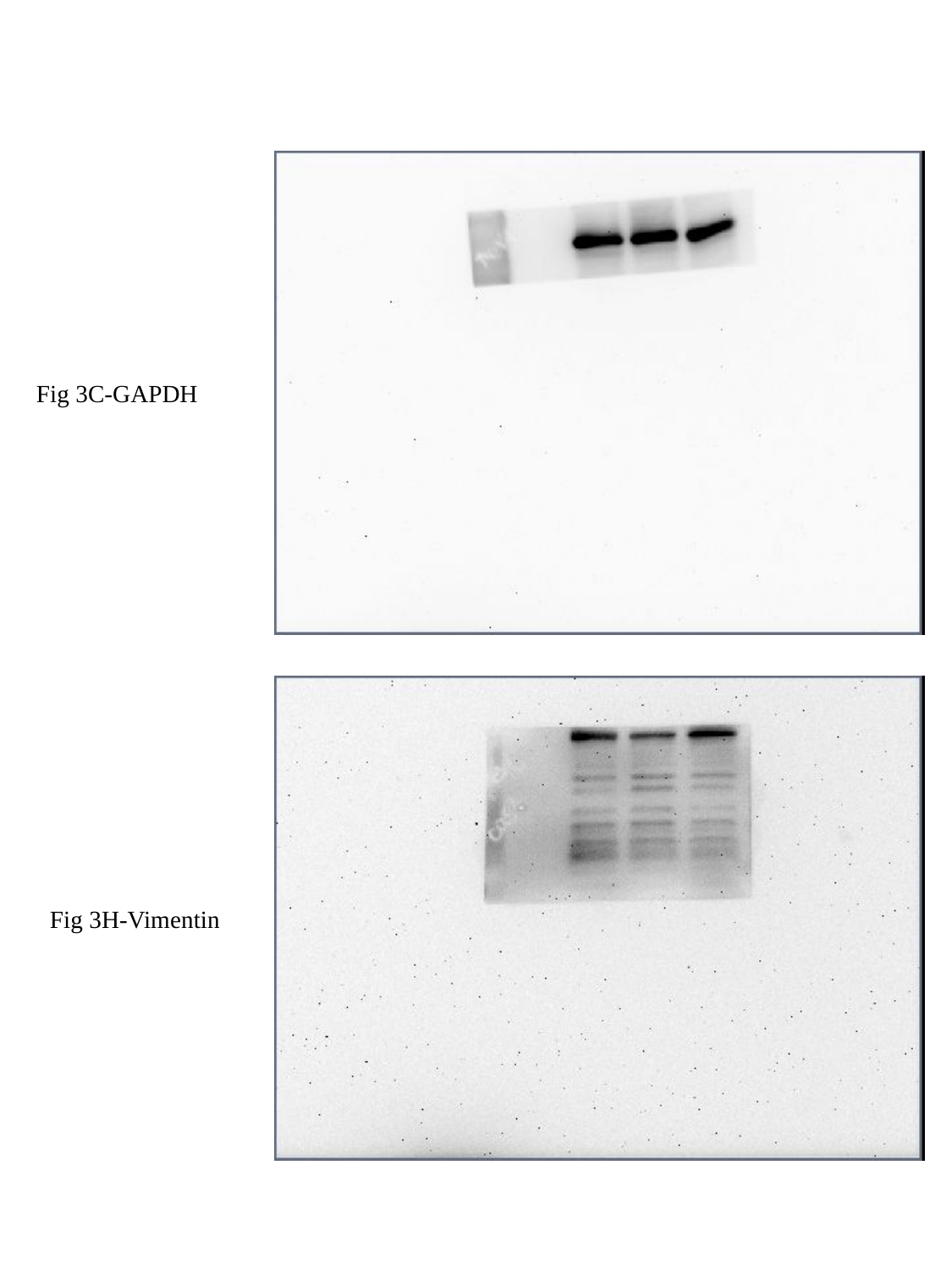

Fig 3C-GAPDH
Fig 3H-Vimentin

## Slide 7
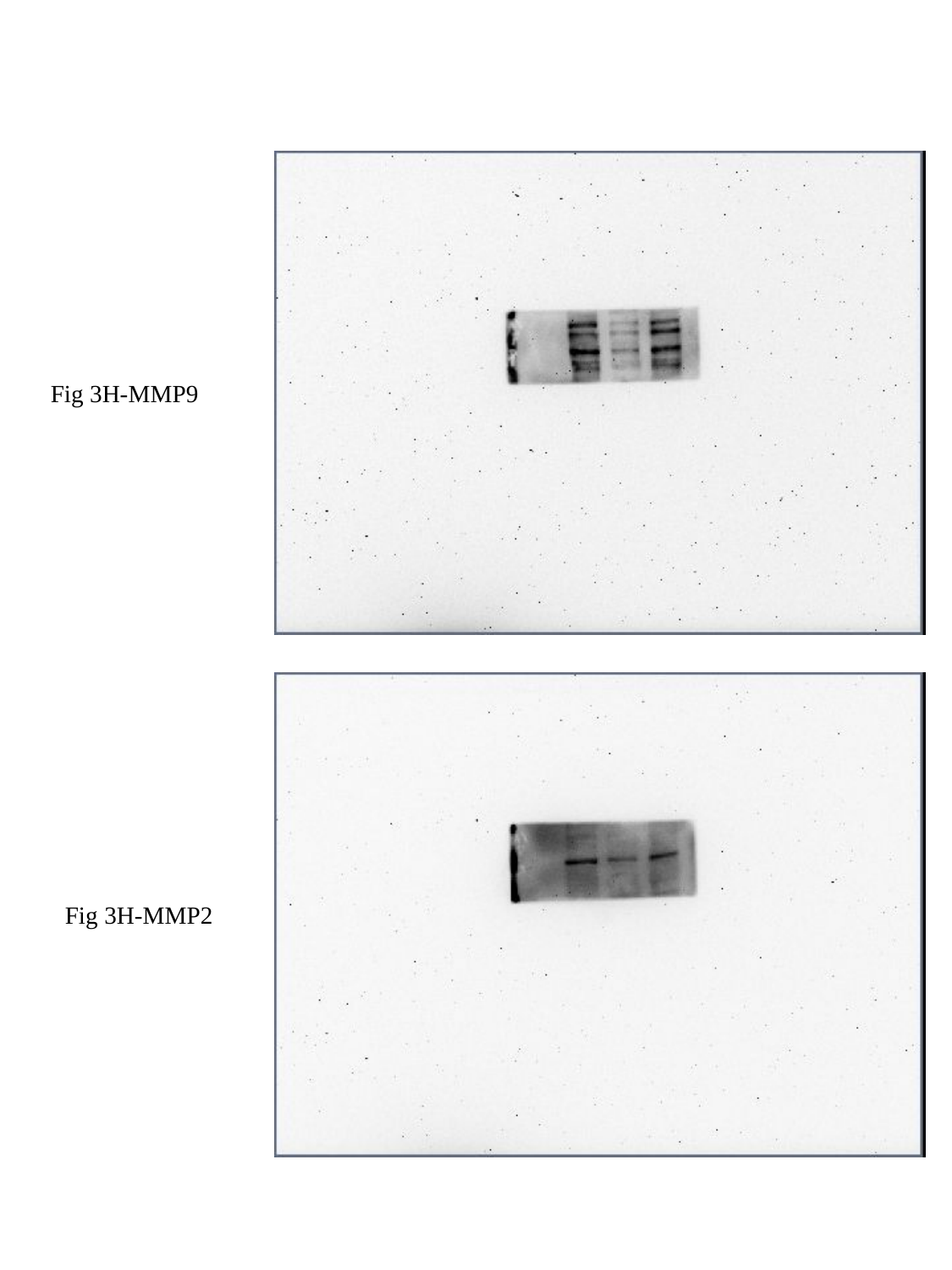

Fig 3H-MMP9
Fig 3H-MMP2

## Slide 8
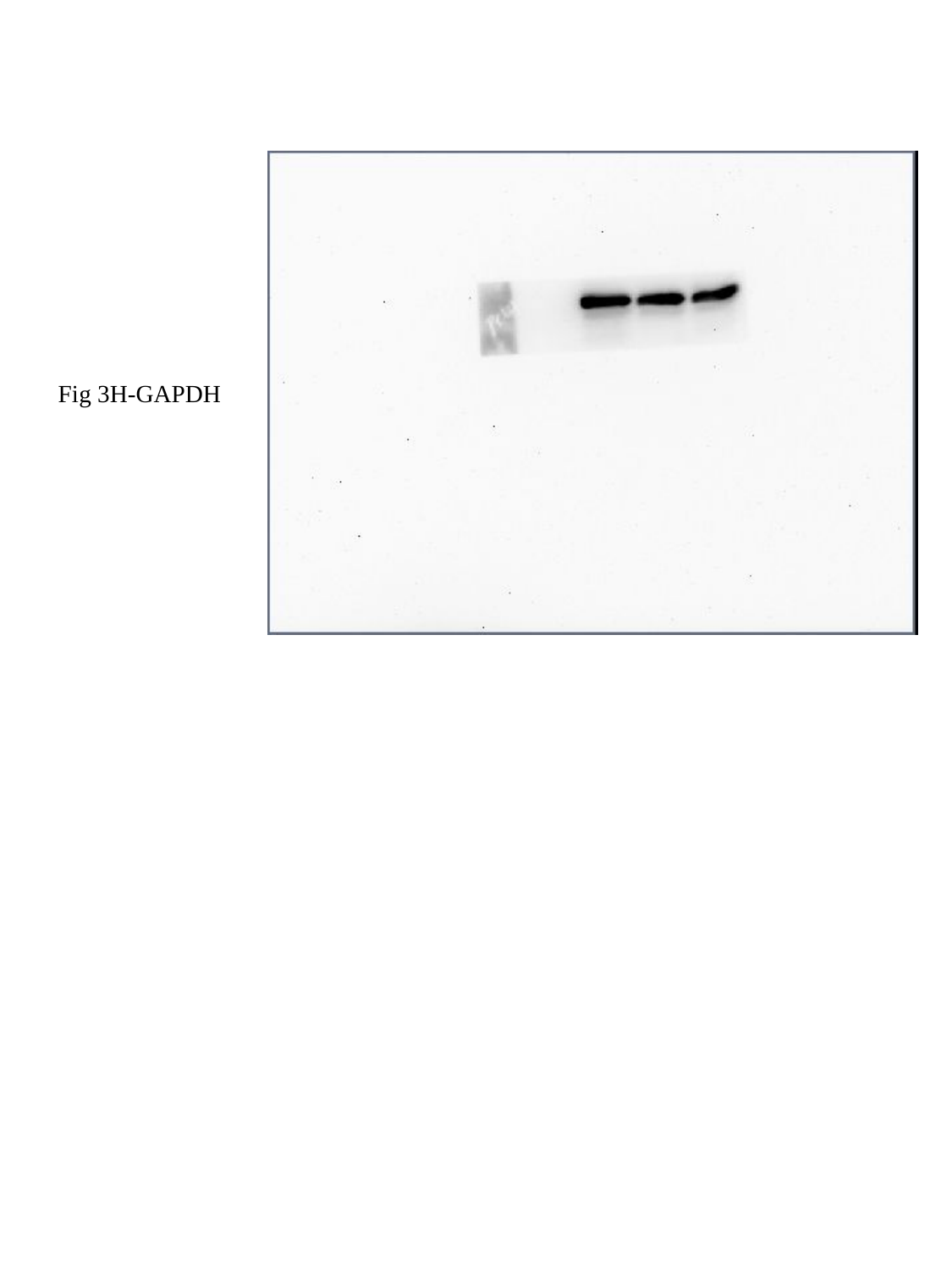

Fig 3H-GAPDH

## Slide 9
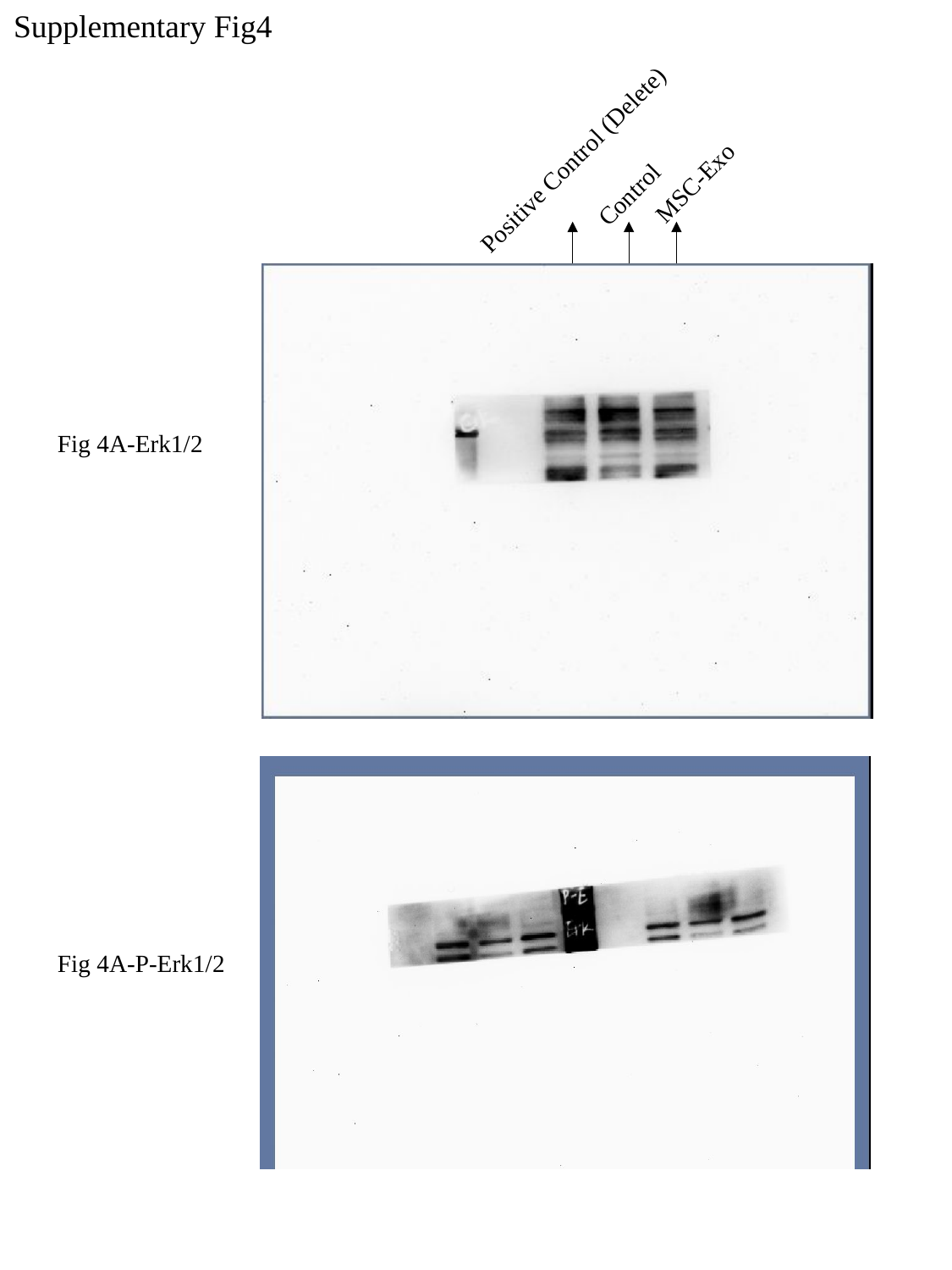

Supplementary Fig4
Positive Control (Delete)
MSC-Exo
Control
Fig 4A-Erk1/2
Fig 4A-P-Erk1/2

## Slide 10
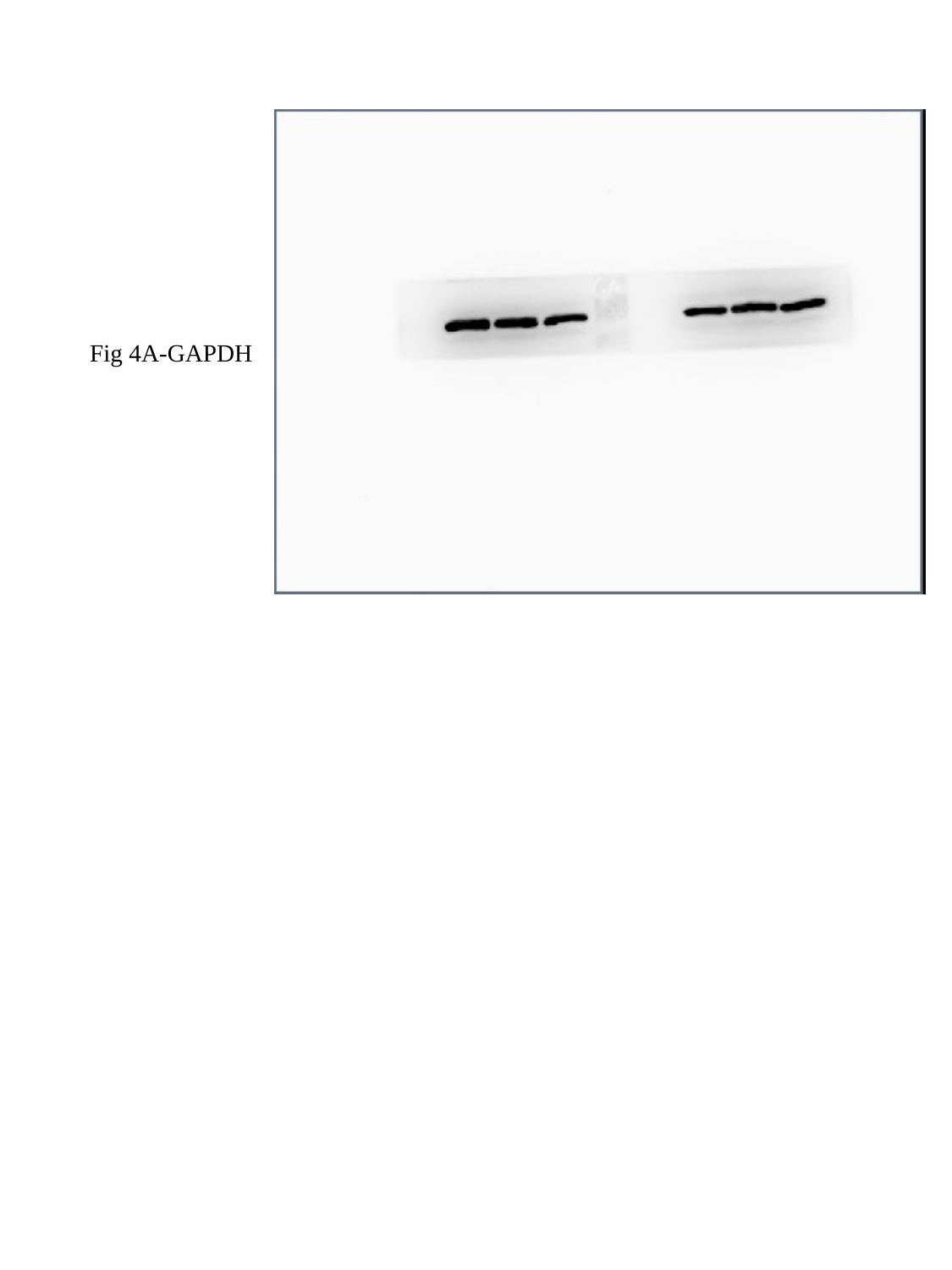

Fig 4A-GAPDH

## Slide 11
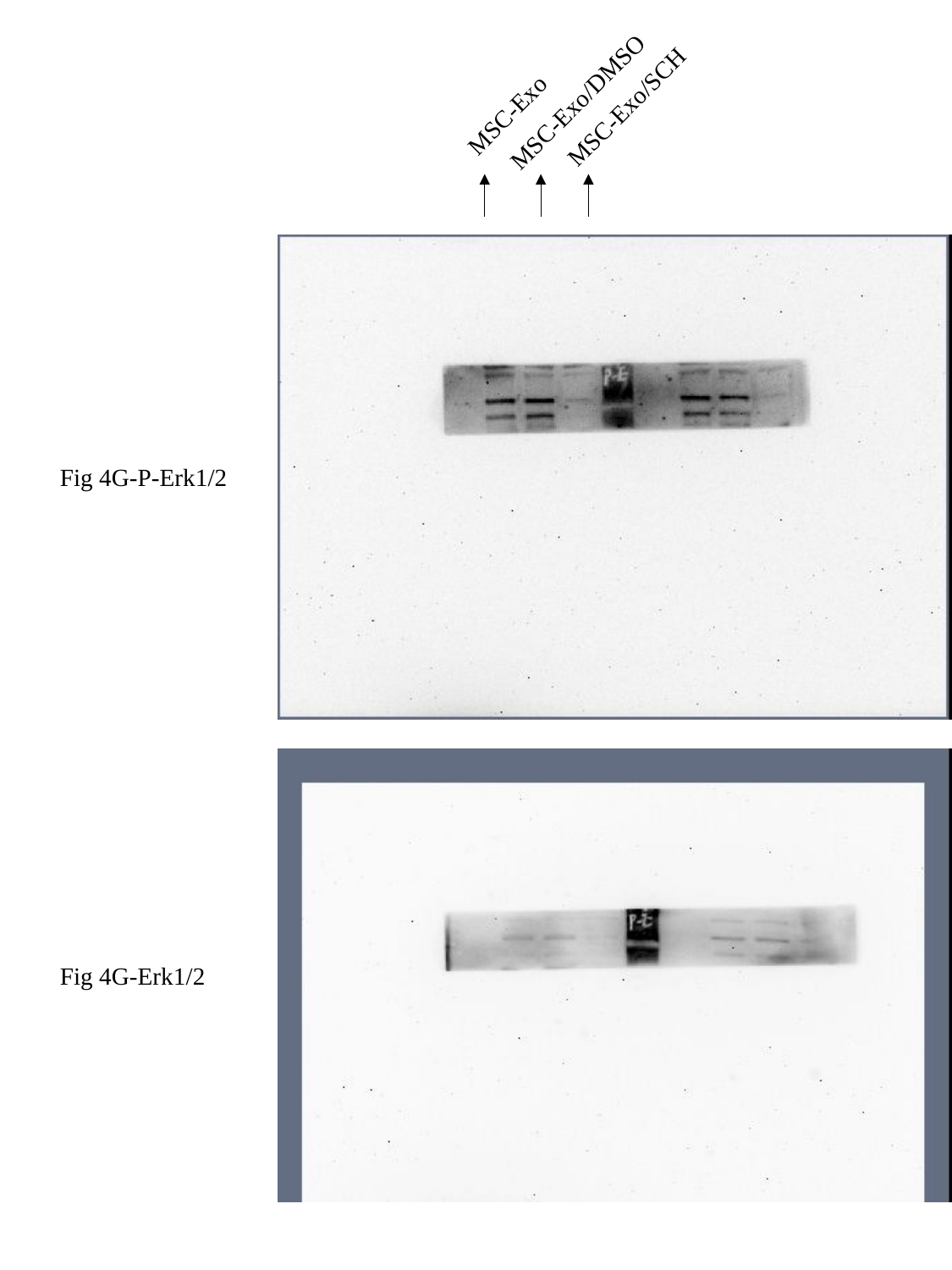

MSC-Exo/DMSO
MSC-Exo/SCH
MSC-Exo
Fig 4G-P-Erk1/2
Fig 4G-Erk1/2

## Slide 12
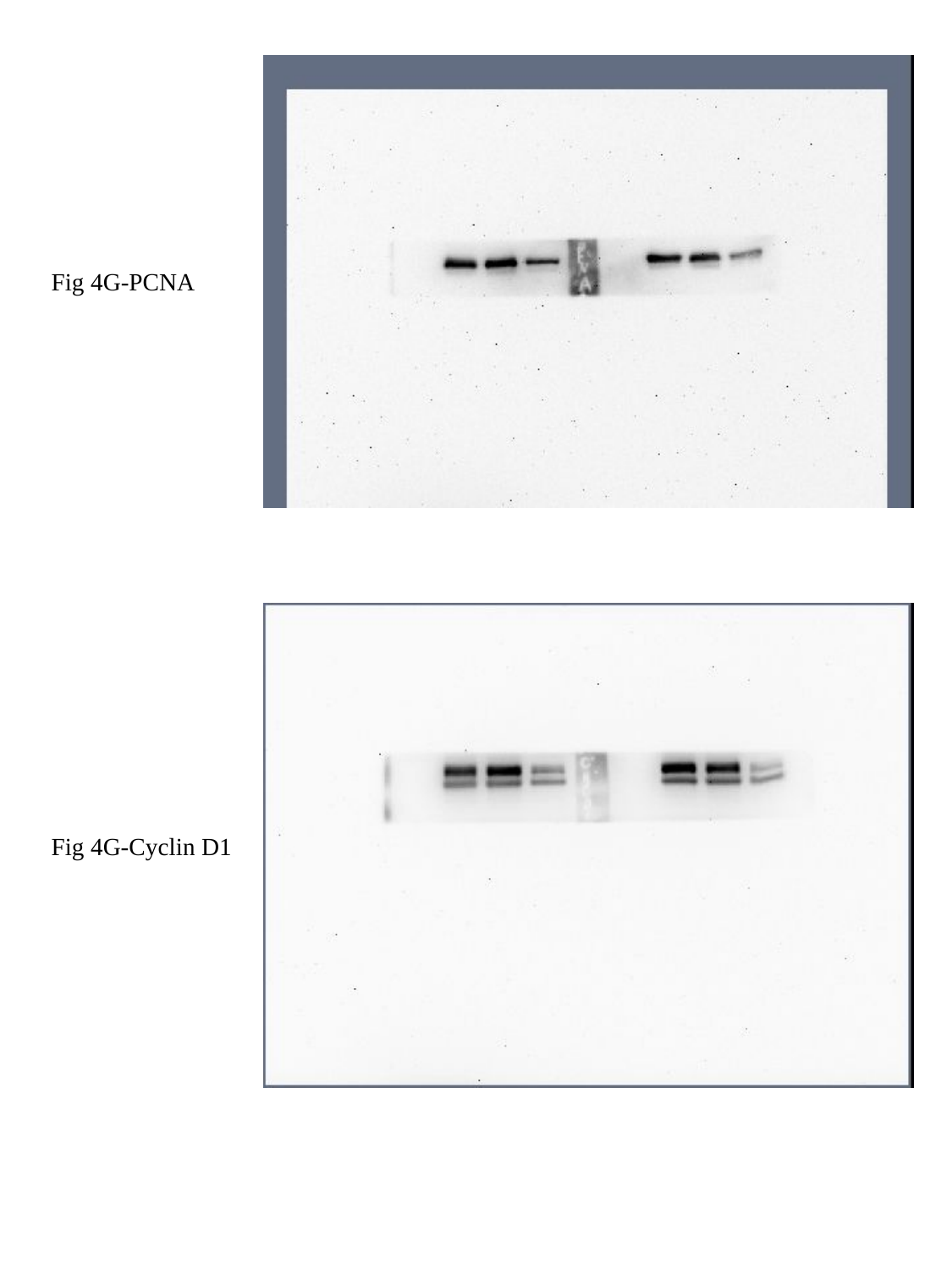

Fig 4G-PCNA
Fig 4G-Cyclin D1

## Slide 13
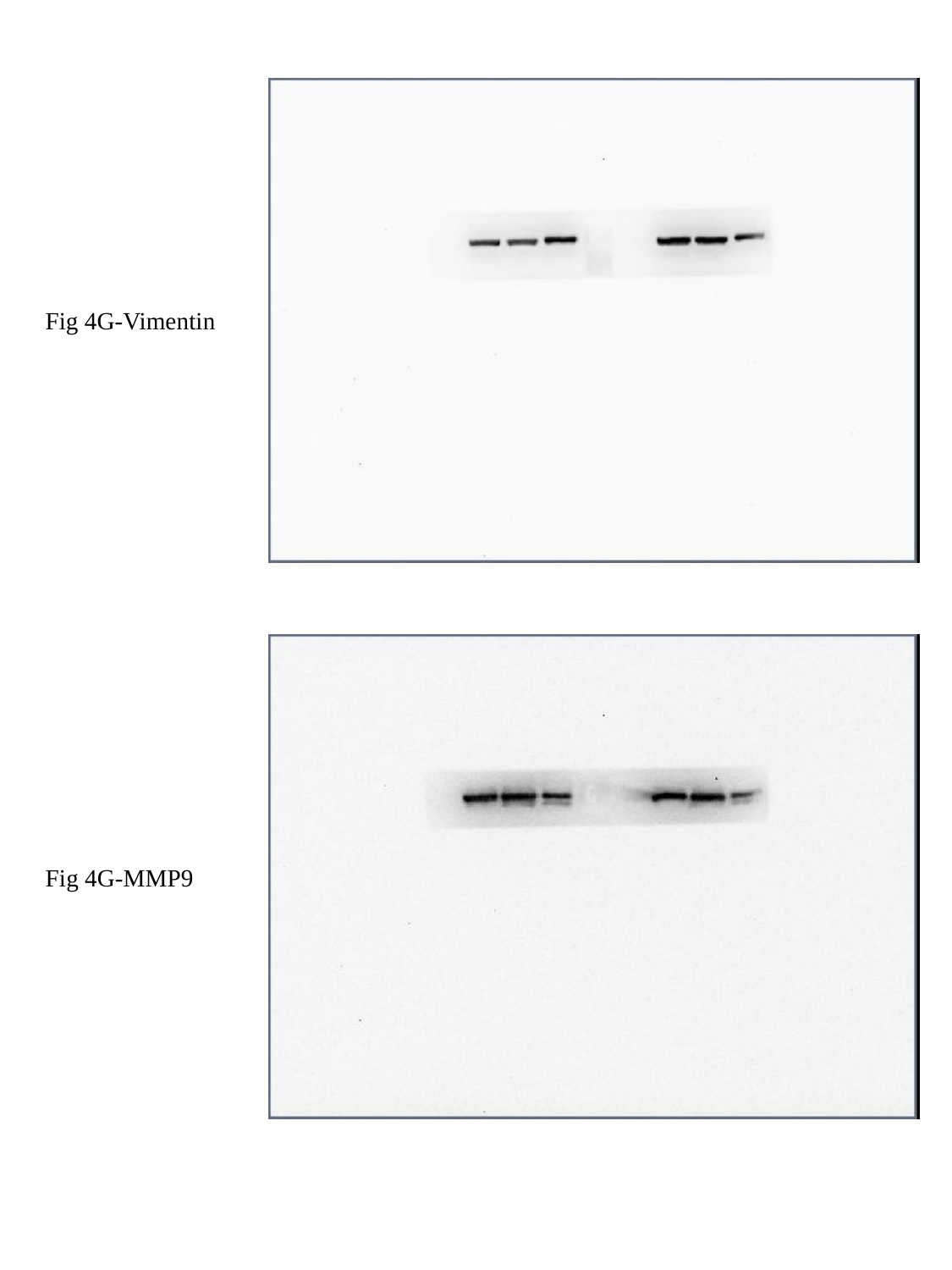

Fig 4G-Vimentin
Fig 4G-MMP9

## Slide 14
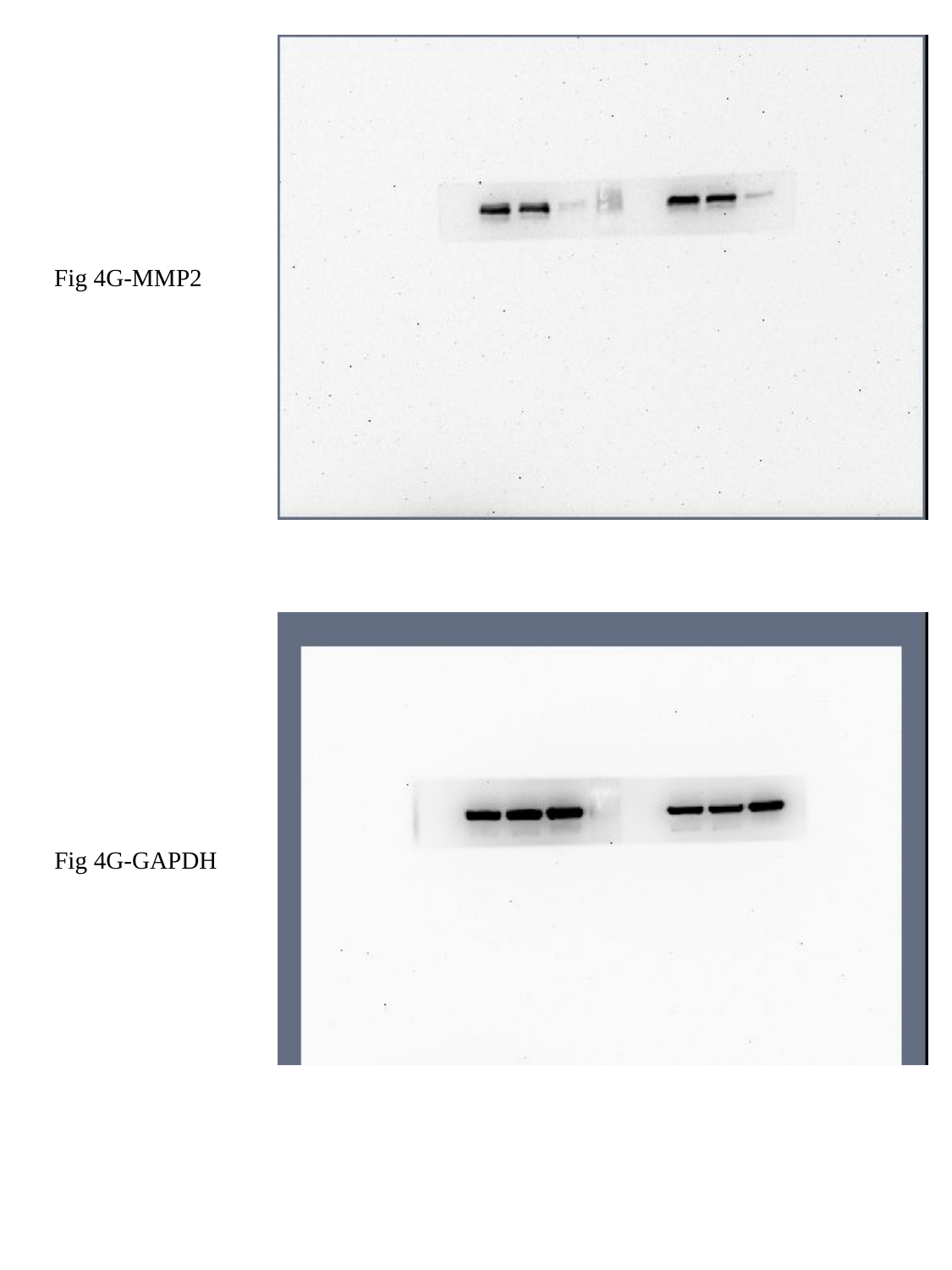

Fig 4G-MMP2
Fig 4G-GAPDH
